# Deep Learning for Schatzker Classification on Anteroposterior Radiographs: A Controlled Benchmark and a Transferable Control Protocol

**DOI:** 10.3390/jcm15187075

**Published:** 2026-09-11

**Authors:** Sang Hyun Na, So Hyun Ahn

**Affiliations:** 1 School of Engineering and Computing, Rice University, 6100 Main St, Houston, TX 77005, USA; sn73@rice.edu; 2 Ewha Medical Research Institute, School of Medicine, Ewha Womans University, Seoul 03760, Republic of Korea; 3 Ewha Medical Artificial Intelligence Center, School of Medicine, Ewha Womans University, Seoul 03760, Republic of Korea

**Keywords:** tibial plateau fracture, Schatzker classification, radiography, deep learning, benchmark controls, confounding by indication, ablation study, shortcut learning

## Abstract

**Background/Objectives**: Schatzker type is assigned early, usually from the anteroposterior (AP) radiograph. A single benchmark accuracy cannot say whether a model read the fracture, the anatomy around it, the annotation, or how the archive was assembled. We ran four inexpensive controls to separate those contributions. **Methods**: We benchmarked a ResNet-50 on PlaTiF, a 2026 public release built for artificial-intelligence research that pairs 421 AP knee radiographs from 186 patients with expert Schatzker labels and per-image tibial segmentations. Evaluation used stratified group five-fold cross-validation grouped by patient, five seeds and balanced accuracy. Inputs were cropped to the expert tibial segmentation shipped with the dataset, an oracle localisation unavailable at deployment. Four controls ran on identical folds: a regression given no pixel content; ablation of the tibial pixels with its complement; a regression on the expert mask alone; and an augmentation audit for label-erasing invariances. **Results**: Among the 128 fracture patients the network reached 0.345 ± 0.030 six-class balanced accuracy, +0.168 over a non-anatomical baseline fitted on the same folds and the same labels (95% CI +0.106 to +0.230, *p* = 0.002). Recall was graded: 0.72 for Schatzker VI, 0.11 for V and 0.04 for IV, the last two below chance (0.167). Erasing the tibial pixels left 0.257 ± 0.025, read on its own as the target bone being unused; its complement, the tibia with everything else removed, reached 0.367 ± 0.012, and the whole radiograph, which carries both, only 0.297 ± 0.032 (+0.071 for the tibia alone, 95% CI +0.031 to +0.111, *p* = 0.008). A regression on the expert mask alone reached 0.213 ± 0.034 and was not distinguishable from the erased model. On fracture versus no classifiable fracture the network reached 0.833 ± 0.028 against 0.814 ± 0.016 for a model given no pixels (*p* = 0.264), and a coronal computed tomography section accompanied 126 of 128 fracture patients but 24 of 58 others (*p* = 2.9 × 10^−19^). **Conclusions**: Each headline number admitted an explanation other than the fracture in the target bone. An ablation reported without its complement misstated where the signal lay, and an augmentation audit overturned our own explanation for the failure of type IV. Controls of this kind cost minutes, and this study illustrates why they can be informative when a benchmark is built on a retrospective clinical archive.

## 1. Introduction

The Schatzker classification sorts tibial plateau fractures into six types and is the vocabulary in which their surgical management is discussed [1,2]. Type determines the approach, the fixation construct and the expected outcome, so it is assigned early, usually from the anteroposterior (AP) radiograph that is the first image a patient receives. Automating that assignment is therefore an attractive target: it would put a consistent reading in front of the clinician at the moment the radiograph appears, before cross-sectional imaging is available.

The classification, however, was not designed for the task it is now asked to perform. Schatzker derived the six types from plain films in 1979, when computed tomography (CT) was not yet routine in musculoskeletal trauma [1]. An AP film is itself a coronal view, and it separates medial from lateral well; what it cannot resolve is everything along the beam. Depth of articular depression, involvement of the posterior column and separation of fragments front to back are all compressed, and the two condylar surfaces are superimposed. The limitation is documented from both directions. Independent observers agree only modestly when they classify from an AP radiograph alone [3], and its original author later revised the system onto CT with a co-author, arguing that plain films cannot support the distinctions the system requires [4].

Where a diagnosis rests on a judgement that is difficult to make by eye, quantitative instrumentation has been brought to bear elsewhere in musculoskeletal medicine: inertial sensors resolve gait asymmetries in lumbosacral discopathy that visual inspection cannot, and their output is interpreted against the imaging that establishes the diagnosis [5]. Supervised Schatzker classification from radiographs has been attempted on private cohorts [6], and the field has until recently had no public dataset on which such a model could be reproduced. That changed in 2026 with PlaTiF [7,8], assembled expressly for artificial-intelligence research: 421 anteroposterior radiographs from 186 patients, each image carrying an expert Schatzker label and a pixel-level tibial segmentation mask, released under an open licence. It is, to our knowledge, the only public collection that pairs this classification with per-image segmentations, which is what makes the anatomical controls used here possible at all. Its descriptor reports no trained model, so the benchmark below is the first on this release.

But a benchmark reports a single number, and a single number cannot distinguish a model that reads the fracture from a model that reads the circumstances under which the image was collected. Retrospective clinical datasets are assembled through a care pathway, and the pathway leaves traces: which patients received which examinations, how the field of view was framed, which annotations were produced. Where the assembly process is correlated with the label, accuracy attributed to image content may belong to the assembly instead. This failure mode is general enough to have a name, shortcut learning [9]. Deep networks have been shown to predict hip fracture largely from scanner and patient process variables rather than from anatomy [10], to select acquisition artefacts over pathology when the two are correlated in the training archive [11], and to lose accuracy when moved to an institution whose pathway differs from the one they were fitted on [12]. A single aggregate score also hides the subgroups in which a model fails, so that a respectable mean can coexist with a class recovered below chance [13]. A systematic review of 62 models built during the COVID-19 pandemic found none fit for clinical use and traced the failures to the same small set of design and reporting omissions rather than to modelling capacity [14]. The controls that separate the two, a non-anatomical baseline fitted on the same partitions, ablation of the target anatomy, and label permutation, are not required by the TRIPOD reporting checklist [15] or its artificial intelligence (AI) extension, and they are not part of standard reporting practice for this task.

This study had two objectives, one clinical and one methodological. The clinical objective was to establish what a convolutional network can and cannot recover of the Schatzker classification from a single AP radiograph, on the first public dataset that permits the question to be asked. The methodological objective was to determine how much of such a benchmark number is attributable to the fracture at all, using four controls stated here in a notation transferable to benchmarks other than this one: a comparator given no pixel content on identical folds, ablation of the target anatomy together with its complement, a regression on the annotator’s mask alone, and an audit of the augmentations for invariances that erase the label. We hypothesised that recovery of the six types from an AP projection would be graded rather than uniform across them and that at least one headline number would admit an explanation other than the fracture in the target bone.

## 2. Materials and Methods

### 2.1. Dataset

We used PlaTiF [7], a public collection of anteroposterior (AP) knee radiographs annotated with the Schatzker classification of tibial plateau fractures, distributed on Zenodo under CC BY 4.0 [8]. The release contains 186 patients from a single academic trauma centre and is distributed under CC BY 4.0. Each patient is stored as one MATLAB MAT-file (v7.3) holding one or more images, indexed im0 to imN. The descriptor lists five elements per image: the original radiograph, a coronal CT section shared by all images of that patient, a binary tibial segmentation mask, a masked tibia image and the Schatzker label.

**Figure 1:**
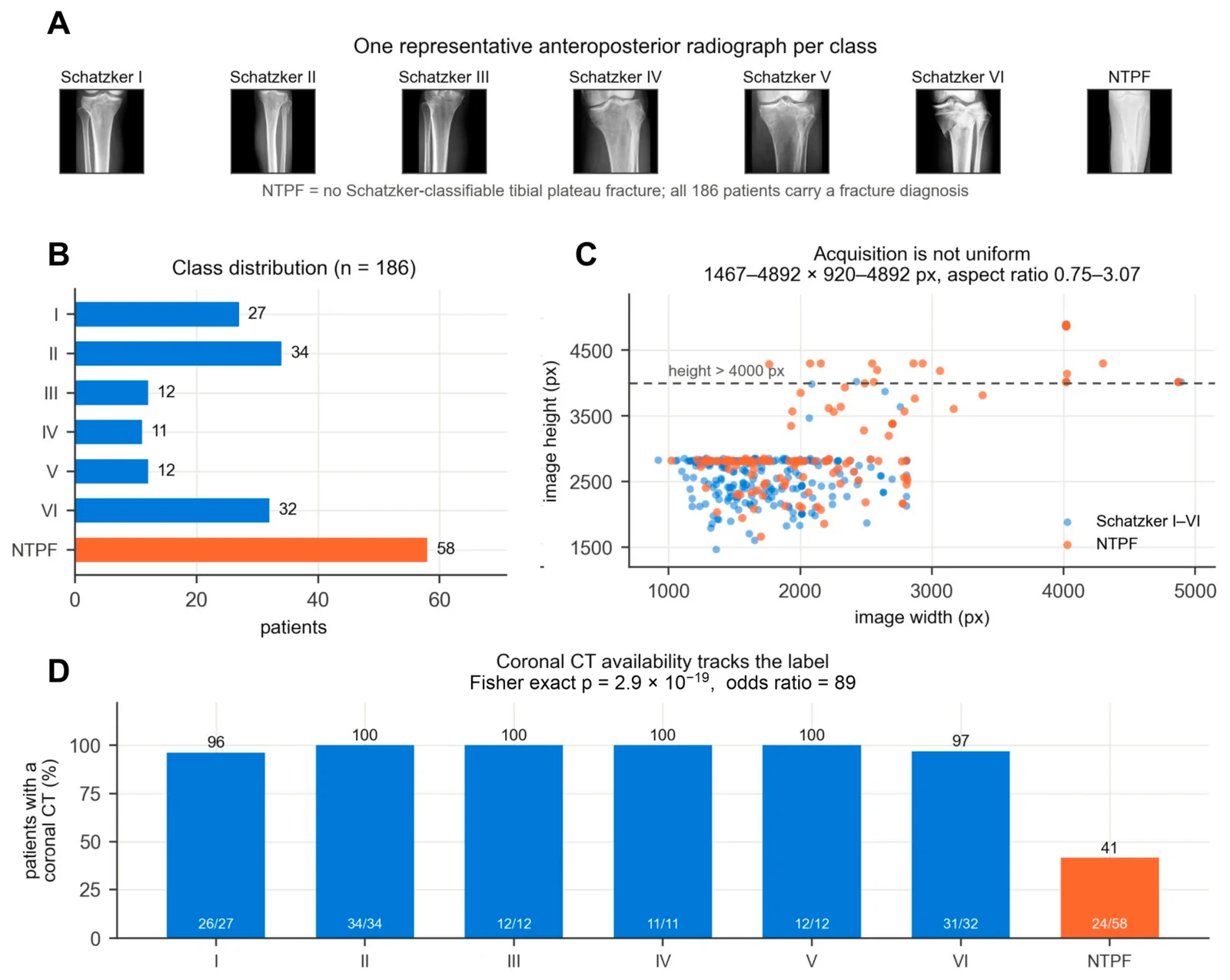
The cohort and the availability of coronal CT. (**A**) One representative anteroposterior radiograph per class, cropped to the bounding box of the tibial segmentation mask distributed with the dataset. NTPF denotes the seventh label of the release, defined as the absence of a Schatzker-classifiable plateau fracture; a fracture diagnosis is recorded for every patient in the released metadata, and the data descriptor defines the class as a normal tibial plateau with or without additional fractures. (**B**) Number of patients per class (*n* = 186). (**C**) Height against width in pixels for all 421 radiographs, coloured by fracture versus NTPF. The dashed line marks a height of 4000 px. Images extending beyond the knee were taken as those with a height above 4000 px or a width above 3000 px, 51 of 421 in total; the dashed line shows the height criterion only. (**D**) Percentage of patients in each class for whom a coronal CT section is present in the release. Counts inside the bars are patients with CT over patients in the class. The contingency table underlying the Fisher exact test collapses Schatzker I to VI into a single fracture group.

Integer labels 1 to 6 denote Schatzker types I to VI. Label 7 denotes the absence of a Schatzker-classifiable plateau fracture, which Table 1 of the data descriptor names No Tibial Plateau Fracture (NTPF); we use that term throughout. NTPF is not a healthy-control class. The released metadata records a fracture diagnosis for all 58 NTPF patients. Fifty-seven are coded as an unspecified fracture of the upper end of the tibia and one as an ankle fracture, and the descriptor defines the class as a normal tibial plateau with or without additional fractures. One representative radiograph of each class is shown in Figure 1A.

### 2.2. Dataset Audit

We parsed all 186 files and recorded, for every image, its dimensions, pixel data type, mask dimensions, mask area and bounding box, and label. Cohort characteristics appear in Table 1 and Figure 1B,C, and the complete per-patient and per-image records are released as Tables S1 and S2.

Five observations from this audit determined how the data were used and are enumerated in Table S6. First, the image-level label disagrees with the metadata spreadsheet for four patients: 230 (I in the image file, IV in the spreadsheet), 235 (VI, I), 238 (IV, I) and 246 (V, IV). Second, for patient 47 image im1 and patient 81 image im1 the mask and the radiograph have different dimensions, so the mask cannot be applied to those two images (Figure S1). Third, the two sources that document the release describe the coronal CT differently: the data descriptor lists it among the elements provided for every image, while the deposit’s own file-structure documentation marks the field optional and present only where available. In the distributed archive it is present for 150 of 186 patients, and its availability is strongly associated with the class (Results). Fourth, the cohort age reported in the descriptor, 45.88 ± 17.54 years with the denominator given as 186 patients, does not reproduce from the released metadata, which yields 43.60 ± 17.85 years; sex counts (149 male, 37 female) and class percentages reproduce exactly. Fifth, four patients (92, 112, 133 and 147) carry different labels across their own images, in every case a fracture type paired with NTPF.

The fifth observation is consistent with bilateral acquisition in a single session, a situation the descriptor Usage Notes anticipates. In three of these patients the images pair by dimension—for patient 92, im0 and im2 are both 2840 × 2534 px and im1 and im3 are both 2850 × 2217 px—while in patient 133 all three images differ in dimension and the pairing is inferred from the label pattern alone. We therefore treated the label as a property of the image rather than of the patient and excluded the five contralateral NTPF images from the six-class task.

Using the image-level label rather than the spreadsheet label changes the composition of the six-class task for the four patients whose labels conflict. That task comprises Schatzker I 26, II 34, III 12, IV 10, V 13 and VI 33 patients, 128 in total, whereas Table 1 and Figure 1B report the spreadsheet labels (I 27, II 34, III 12, IV 11, V 12, VI 32).

### 2.3. Preprocessing and Model Inputs

Pixel intensities in the release are stored as double precision in the interval [0, 1]; we clipped to that interval and mapped it linearly onto 8 bits. Images were resampled to 448 × 448 px preserving aspect ratio, with centre padding by zeros. Writing s for the linear scale ratio applied to an image, s = min(448/height, 448/width), the median value of s is 0.159 and the minimum 0.092, so bilinear interpolation alone aliases the high-frequency content that carries cortical discontinuity. We therefore applied a Gaussian prefilter with standard deviation (1/s − 1)/2 pixels before interpolation whenever that quantity exceeded 0.05.

**Figure 2:**
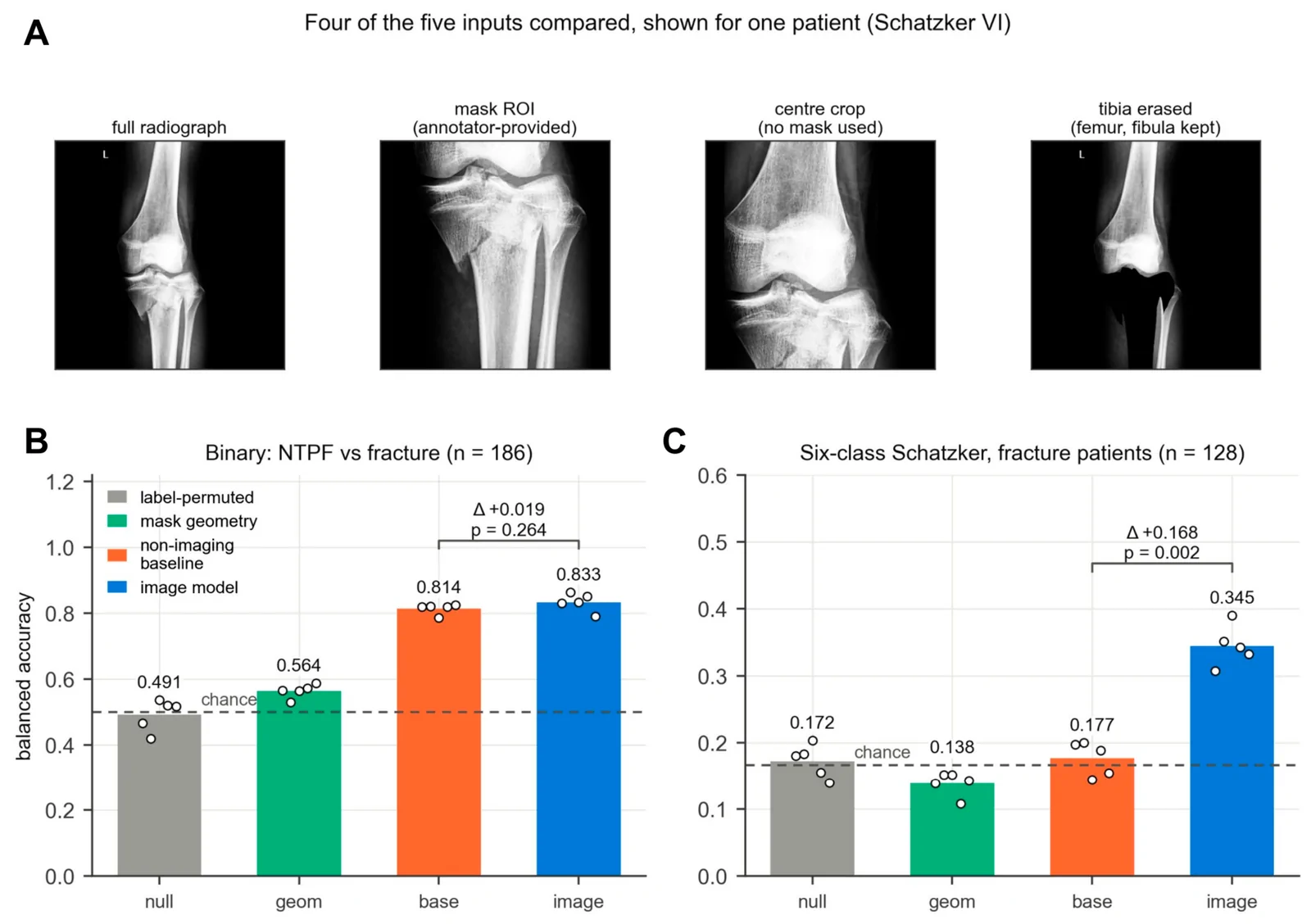
Model inputs, and balanced accuracy on the two classification tasks. (**A**) One Schatzker VI patient rendered as four of the five inputs prepared in this study, three of which are evaluated in this panel: the full radiograph; the mask ROI, that is the bounding box of the distributed tibial mask expanded by 12%; the centre crop, a box of the same size placed at the image centre without using the mask; and the tibia-erased input, in which pixels inside the tibial mask are set to zero while the femur, fibula, joint space and soft tissue are retained. (**B**) Binary task, NTPF versus fracture, *n* = 186 patients. (**C**) Six-class Schatzker typing among the 128 fracture patients. In both panels the bars are, left to right, the label-permuted null, logistic regression on mask geometry alone, the prespecified non-anatomical baseline, and the ResNet-50 on mask-ROI inputs; bar height is the mean over five seeds and the open circles are the individual seeds. All four predictors use identical patient-level partitions. The dashed line is chance. The bracket gives the paired difference between the image model and the prespecified baseline, with the *p* value of a paired *t*-test across seeds.

Five inputs were prepared from each radiograph (Figure S2) and all five were evaluated under the five-seed protocol; four of them, excluding tibia-only, are also shown in Figure 2A. They differ in field of view as well as in content, and the comparisons below are interpreted accordingly.

- **Mask ROI (region of interest)**—the bounding box of the tibial mask distributed with PlaTiF, expanded by a 12% margin, cropped from the radiograph.
  - **Full**—the entire radiograph, uncropped.
  - **Tibia-erased**—the entire radiograph with the pixels inside the tibial mask set to zero. Since the distributed mask segments the tibia alone, the femur, fibula, joint space and soft tissue remain. The erased region retains the outline of the mask, including the contour of the tibial plateau.
  - **Tibia-only**—the exact complement of the tibia-erased input: the entire radiograph with every pixel outside the tibial mask set to zero, so that the tibia is retained and the femur, fibula, joint space and soft tissue are removed. Tibia-erased and tibia-only share a field of view and partition the radiograph between them, which makes their contrast a single-variable one.
  - **Centre crop**—a box of the same size as the mask ROI placed at the image centre, cropped from the radiograph without using the mask.

The mask ROI is an oracle localisation. It uses a segmentation produced by the dataset’s annotators and supplied at inference time, which would not be available in a deployed system; we trained no segmentation model. Results on this input therefore bound what a classifier can achieve given perfect localisation rather than what an end-to-end pipeline would achieve. The full input is shown in Figure 2A and Figure S2 for reference and was not evaluated under the five-seed protocol described below.

### 2.4. Model and Training

We fine-tuned ResNet-50 [16] initialised from the torchvision v0.19.1 IMAGENET1K_V2 weights, replacing the final fully connected layer. The 448 × 448 input is accommodated by the network’s adaptive average pooling. The single grey channel was replicated three times and normalised with mean 0.449 and standard deviation 0.226, the grey-scale equivalent of the ImageNet statistics. Optimisation used AdamW with weight decay 10^−4^ under a one-cycle schedule with maximum learning rate 3 × 10^−4^ and the default warm-up fraction of 0.3, batch size 16, mixed precision, and class-weighted cross-entropy with label smoothing 0.05, for a fixed 25 epochs. The class weights were derived from the number of patients in each class but applied to a loss taken over images, so a patient contributing eight images carries eight times the weight of a patient contributing one. Evaluation is at patient level regardless. We repeated the six-class mask-ROI configuration with every image weighted by the reciprocal of the number of images its patient contributes, so that each patient carries the same weight in training as in evaluation, and report it in Section 3.5. Augmentation was deliberately light: horizontal flip with probability 0.5, brightness scaling between 0.9 and 1.1 with probability 0.5, and translation of up to ±12 px with probability 0.3. Horizontal flipping makes the network invariant to the mirror image of its input. The whole image is mirrored together, so the fracture keeps its position relative to the fibula and the condyles and the anatomical medial–lateral distinction survives the operation; what the operation removes is the side of the frame on which a feature appears, and the mask ROI additionally excludes the radiographic laterality marker. Whether that invariance nonetheless accounts for the behaviour of Schatzker IV is taken up in the Results.

To test that consequence we repeated the six-class mask-ROI configuration with horizontal flipping disabled and everything else held fixed. That run used a reimplementation that applies the augmentations on the GPU, because contention for the host CPU on the shared machine had made the original data-loading path the bottleneck; the partitions, seeds, epochs, batch size, optimiser, schedule, loss and the probabilities and ranges of the remaining augmentations are unchanged, and only the device on which the operations run differs. To keep the contrast to a single variable we also reran the flip-on configuration through that same reimplementation. All three runs use identical patient-level folds, verified row by row on the (seed, image, fold, label) tuples.

We performed no hyperparameter search, used no validation split and applied no early stopping. This protocol was fixed in advance. It removes optimism arising from model selection; in exchange the accuracies reported here are a lower bound on what a tuned configuration could reach, and convergence has to be demonstrated separately, which the training-accuracy and label-permutation controls provide.

Every source of randomness was seeded, including the Python (v3.11.10), NumPy (v1.26.3) and PyTorch (v2.4.1) generators, CUDA, cuDNN deterministic mode, and the data-loader generator and worker initialisation. Each configuration was run with five seeds, 0 to 4.

### 2.5. Evaluation and Statistical Analysis

All models were trained and evaluated with stratified group five-fold cross-validation, using the patient as the grouping unit and the seed as the cross-validation random state, so that every image of a patient falls on the same side of every partition. The folds are formed over images, so the stratification balances the number of images in each class rather than the number of patients. A patient contributes up to eight images and the label is a property of the limb, so splitting at the image level would place images of the same limb on both sides of the partition. Evaluation is also at patient level: the per-image logits of a patient are averaged, giving one vote per patient. For the binary task the patient-level reference label is that of the patient’s first image; all four patients with mixed image-level labels have a fracture image first, so this rule and the rule “fracture if any image carries a Schatzker label” agree for every patient in the cohort. No held-out test set and no external cohort were available. Every value reported here is an internal cross-validated estimate within the same 186 patients of a single centre and is not a generalisation estimate.

Balanced accuracy is the primary metric and macro-F1 is reported alongside it. Class prevalence makes unweighted accuracy uninformative: in the binary task a majority-class predictor reaches 0.688 accuracy, and in the six-class task the largest class alone reaches 0.266.

Two kinds of uncertainty are reported and they are not interchangeable. The seed sets the cross-validation random state as well as the training randomness, so each seed draws a different partition of the same 186 patients; all models compared at a given seed share that seed’s partition exactly, which is what makes the comparisons paired. Seed-level standard deviations therefore combine training randomness with the redrawing of the partition, and with five seeds the paired tests have four degrees of freedom and little power. The seed is the unit of replication because it redraws the partition while every model compared at that seed shares it, which is what makes the comparisons exactly paired; fold-level values are not used for inference because the folds of one seed are not independent of one another. Five seeds were fixed in advance as part of the protocol described in Section 2.4, and the number is a compute constraint rather than a statistical choice: the grid actually run is thirteen configurations by five seeds by five folds, that is, 325 fine-tuning runs, and each additional seed adds thirteen more. Because the paired tests have four degrees of freedom we do not read a non-significant seed-level difference as evidence of equivalence, and uncertainty about which patients the cohort contains is carried by the patient-level bootstrap described next rather than by the seed tests. Uncertainty arising from which patients are in the cohort is given separately, as a stratified bootstrap 95% interval from 20,000 resamples in which the resampling unit is the patient: patients are drawn with replacement within a class and all five of a drawn patient’s evaluations enter together, so the interval respects the dependence between repeated evaluations of one patient. Intervals obtained by resampling evaluations rather than patients are 1.4 to 1.9 times narrower for the model and baseline comparisons and are not reported; for the label-permutation controls the two schemes give intervals of essentially the same width. Per-class recall is reported with a patient-level cluster bootstrap interval from 20,000 resamples in which patients are drawn with replacement within a class and all five evaluations of a drawn patient enter together, so that the interval reflects the repeated evaluation of the same patients. Clopper–Pearson exact intervals [17] computed over the pooled evaluations are tabulated alongside them in Table S5; their denominators are five times the number of patients in the class and they do not account for the dependence, which makes them 1.3 to 1.6 times narrower than the clustered intervals in Schatzker I, II, III and VI. In types IV and V the clustered interval is instead the narrower of the two, because almost every patient of those classes is misclassified under every seed and the resampled distribution is close to degenerate; for those two classes we report the distribution of per-patient outcomes rather than relying on either interval. All tests are two-sided, and no correction for multiple comparisons was applied because a single primary comparison was specified in advance.

### 2.6. A Common Notation for the Four Controls

The four controls are stated here in one notation so that they can be applied to a benchmark other than this one. Write each patient as (x, z, M, y): x is the radiograph, z is the non-image covariates recorded alongside it, M is the binary tibial mask distributed with the release, and y is the Schatzker label. Let P denote a patient-level partition into five folds, let m(x, M) be the radiograph with every pixel outside M set to zero, and let grid(M) be a coarse binary grid of the mask alone, carrying no radiograph pixel. Write

A(h, T; P)

for the balanced accuracy of a model class h trained and evaluated over P on the input transform T. Every comparison below holds P fixed and is paired seed by seed, so a difference between two rows is a difference in T or in h and not in the partition. The partitions the convolutional models used were recovered from their out-of-fold prediction files and reused verbatim for every non-convolutional comparator, which is what makes the pairing exact.

C1, identifiability. Δ = A(f, x; P) − A(g, z; P). If Δ is not distinguishable from zero, an accuracy computed on x cannot be attributed to the image, because a model that never sees x reaches the same value.

C2, complementary ablation. A(f, m(x, M); P), A(f, m(x, 1 − M); P) and A(f, x; P). The two masked inputs partition x, so reporting either one alone leaves the location of the signal unresolved; the direction of the ablation can invert its own reading.

C3, annotation alone. Δ = A(f, m(x, 1 − M); P) − A(g, grid(M); P). Setting the interior of M to zero leaves its boundary in place, so this separates what the annotator drew from the anatomy it outlines.

C4, invariance audit. For the augmentation group G applied during training, whether some element of G maps between label classes, which would remove information the label depends on.

None of the four requires access to model weights, and each costs minutes once the benchmark itself has been run. Section 2.7, Section 2.8 and Section 2.9 give the concrete instantiation used here.

### 2.7. Non-Anatomical Baseline

That primary comparator is a logistic regression on non-anatomical metadata. It is given no pixel content, but six of its features (maximum image height, maximum image width, the larger of the two, the whole-leg and landscape flags, and the mask area fraction) are derived from the images and their masks rather than from the clinical record. We therefore call it non-anatomical rather than non-imaging: what none of its features describes is anatomy. Its features are age, laterality, the number of images available for the patient, coronal CT availability, maximum image height, maximum image width, the larger of those two, presence of a whole-leg view, presence of a landscape-orientation image, and the mask area fraction averaged over the patient’s images; laterality is encoded as three indicators, giving twelve columns. That last feature is the mean over a patient’s images, not the per-patient median tabulated in Table S1. Features were standardised and the model fitted with L2 regularisation at the scikit-learn default strength, balanced class weights and a maximum of 2000 iterations. It was fitted and evaluated on the same (seed, fold) patient partitions as the image models and against the same image-level patient labels, so the two are paired seed by seed.

Variants using mask geometry alone or both feature sets, and a random-forest alternative, are reported in Table S4 and were not the prespecified comparator.

### 2.8. Mask Silhouette Control

Because the tibia-erased input retains the outline of the annotator’s mask, we measured how much Schatzker information that outline carries on its own. For each of the 419 images with an applicable mask we reduced the binary mask to a coarse grid, preserving aspect ratio with centre padding. The regression was then fitted on the 275 images of the 128 fracture patients that enter the six-class task, using the same standardised logistic model as the non-anatomical baseline and the identical (seed, fold) partitions, so roughly 220 images train each fold. No radiograph pixel enters this model; its only input is the shape, position and extent of the mask within the frame. We report two grid sizes, 16 × 16 and 48 × 48. Neither was specified in advance, this control was not part of the prespecified comparison, and its *p* values are not corrected for the two resolutions examined.

### 2.9. Attribution

Gradient-weighted class activation mapping (Grad-CAM) maps [18] were computed at the last residual block of the mask-ROI six-class model, for the test patients of fold 1 of seed 0, and normalised to unit maximum. To summarise them we measured the fraction of Grad-CAM mass falling in the outer 15% margin of the input. That margin occupies 0.509 of the image area, so a spatially uniform map would yield 0.509 by construction. Because several images may come from one patient, the maps were averaged within a patient before testing. This attribution describes the mask-ROI model only; no Grad-CAM was computed for the tibia-erased model. Because the maps come from a single fold of a single seed and from 24 patients, we treat this analysis as exploratory throughout and draw no conclusion of this study from it.

### 2.10. Software and Availability

Analyses used Python v3.11.10 with NumPy v1.26.3, SciPy v1.11.4, PyTorch v2.4.1 [19] and scikit-learn v1.9.0 [20]. Balanced accuracy and macro-F1 follow the scikit-learn definitions, Fisher exact tests used SciPy, and Clopper–Pearson intervals [17] were computed from the beta quantile function. The processed per-patient and per-image tables that support every analysis are released as Tables S1 and S2. Code is available at https://github.com/sanghyunn82-creator/platif-controlled-benchmark (accessed on 8 September 2026).

## 3. Results

### 3.1. Cohort and Acquisition

The 186 patients contributed 421 AP radiographs, one to eight each (median two), spanning all six Schatzker types and the NTPF class (Figure 1A). Age was 43.60 ± 17.85 years (range 15–88) and 149 of 186 patients were male. The fractured side was recorded as right in 96 patients, left in 87 and bilateral in three (Table 1). Class membership was uneven: NTPF 58, Schatzker II 34, VI 32, I 27, III 12, V 12 and IV 11 by the metadata spreadsheet (Figure 1B); the image-level labels used for modelling redistribute four of these patients, as the Materials and Methods describes.

Acquisition was not standardised. Image height ranged from 1467 to 4892 px and width from 920 to 4892 px, with aspect ratios from 0.75 to 3.07. Fifty-one of four hundred and twenty-one images extended beyond the knee, taken as a height above 4000 px or a width above 3000 px (Figure 1C).

### 3.2. Coronal CT Availability Is Confounded with the Label

A coronal CT section accompanies 126 of 128 fracture patients but only 24 of 58 NTPF patients (Fisher exact *p* = 2.9 × 10^−19^, odds ratio 89; Figure 1D). Availability is 96–100% in every fracture class and 41% in NTPF. The single rule “no coronal CT implies NTPF” reaches a balanced accuracy of 0.785 on its own (*n* = 186 patients).

This variable records the care pathway rather than the anatomy: a coronal CT is obtained when a plateau fracture is suspected. The convolutional network never sees it, so it is not a shortcut available to the model. What follows from this for the interpretation of any task that includes the NTPF class is taken up in Section 4.3.

### 3.3. On the Binary Task the Image Model Does Not Separate from a Non-Anatomical Baseline

We compared four predictors of NTPF versus fracture on identical patient partitions (Figure 2B, Table 2). Permuting the labels gave 0.491 ± 0.048. Mask geometry alone gave 0.564 ± 0.021. The prespecified non-anatomical baseline reached 0.814 ± 0.016 and the ResNet-50 on mask-ROI inputs reached 0.833 ± 0.028, with a patient-cluster bootstrap interval of 0.782 to 0.882 (*n* = 186 patients, five seeds). The paired difference between them is +0.019 (95% CI −0.022 to +0.060, *t* = 1.30, df = 4, *p* = 0.264); resampling patients rather than seeds gives +0.019 (95% CI −0.018 to +0.056).

The two are therefore not distinguishable in this cohort. With five seeds the paired test has four degrees of freedom and little power, so this is a failure to distinguish and not evidence of equivalence; Section 4.9 states what the comparison does and does not resolve.

### 3.4. Among Fracture Patients the Image Model Does Separate

Restricting the analysis to the 128 fracture patients removes the confound, because a coronal CT is present for 126 of them and the variable is close to constant. The non-anatomical baseline falls to 0.177 ± 0.026, just above a chance level of 0.167, while the image model reaches 0.345 ± 0.030 (macro-F1 0.335 ± 0.031; bootstrap interval 0.301 to 0.389; Figure 2C). The paired difference is +0.168 (95% CI +0.106 to +0.230, *t* = 7.49, df = 4, *p* = 0.002; *n* = 128 patients, five seeds) and +0.168 (95% CI +0.125 to +0.210) when patients rather than seeds are resampled. The comparator pools twelve columns of three provenances, and fitting each provenance separately on the same partitions shows that it is not the strongest non-anatomical predictor these data allow: against the strongest block the image model leads by +0.093 (95% CI +0.049 to +0.137, *p* = 0.004). The blocks were separated after the main results were known and are reported as a sensitivity analysis rather than as a replacement for the comparator fixed in advance (Table S7).

### 3.5. Erasure and Its Complement

**Figure 3:**
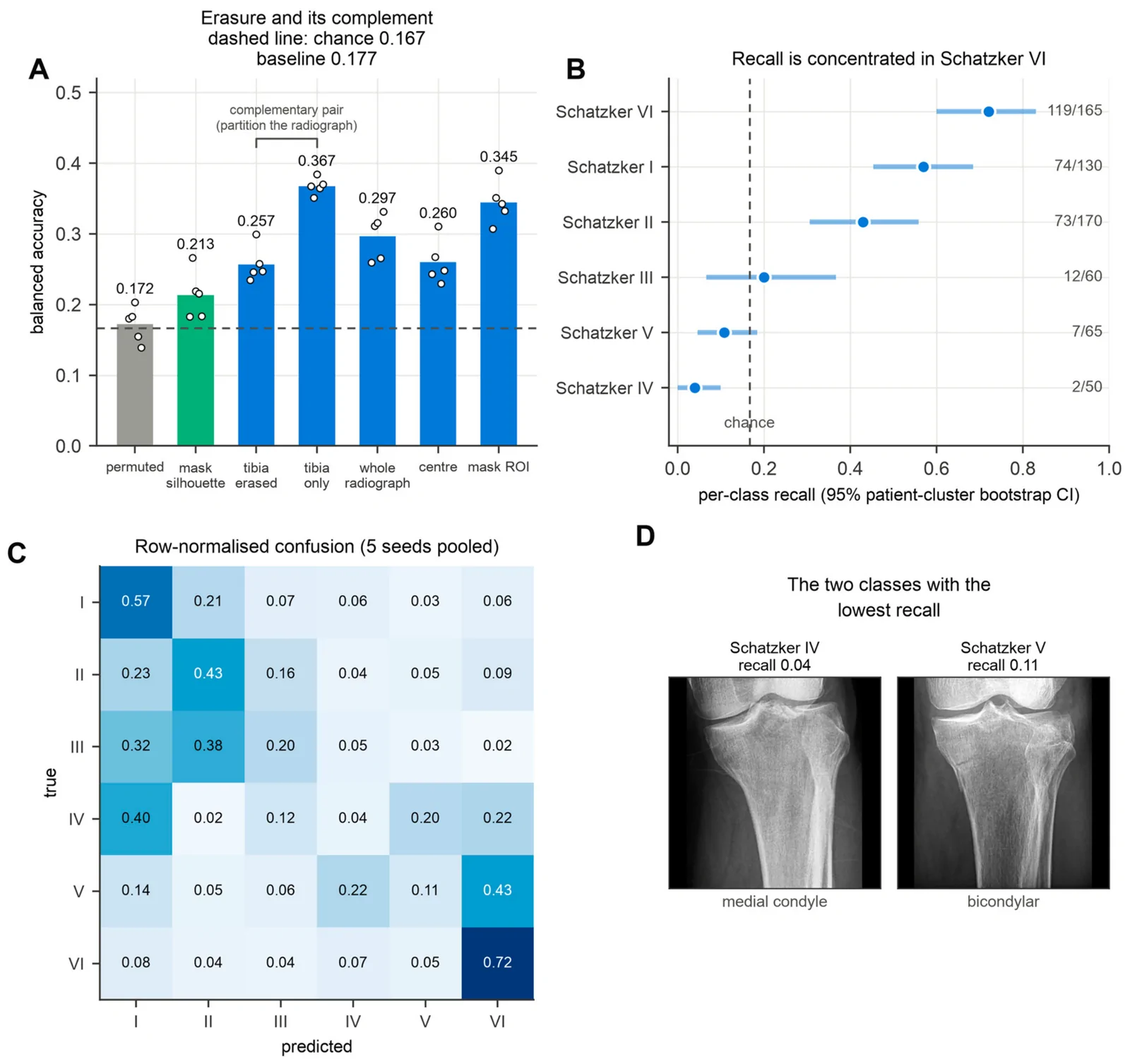
Where the six-class model fails. (**A**) Balanced accuracy of the six-class task under seven conditions, five seeds each: labels permuted, a logistic regression on the annotator’s mask silhouette with no radiograph pixels, and the network on tibia-erased, tibia-only, whole-radiograph, centre-crop and mask-ROI inputs. Bars are the mean over seeds and open circles the individual seeds; the number above each bar is the mean. The bracket marks the two inputs that are exact complements of one another and share a field of view, so that between them they partition the radiograph. The dashed line marks chance; the prespecified non-anatomical baseline sits just above it, at 0.177. (**B**) Per-class recall with 95% patient-level cluster bootstrap intervals, 20,000 resamples with the patient as the resampling unit, pooled over five seeds; the count at the right of each interval is correct patient evaluations over total patient evaluations, so that each of the 128 patients contributes five times. The dashed line is chance. (**C**) Row-normalised confusion matrix for the mask-ROI model, five seeds pooled; rows are true classes and columns predicted classes; cell shading is proportional to the value printed in the cell. (**D**) Mask-ROI radiographs of one Schatzker IV and one Schatzker V patient, the two classes with the lowest recall; recall for these two types is given in panel (**B**).

We repeated the six-class experiment with the pixels inside the tibial mask set to zero. That model reached 0.257 ± 0.025 (bootstrap interval 0.211 to 0.305), above the baseline by +0.080 (95% CI +0.022 to +0.139, *t* = 3.80, df = 4, *p* = 0.019; Figure 3A). Restoring the tibial pixels adds a further +0.088 (95% CI +0.073 to +0.102, *t* = 16.6, df = 4, *p* = 0.0001; patient-cluster bootstrap +0.088, 95% CI +0.043 to +0.132).

That second comparison changes the field of view as well as the content, so the +0.088 bounds the joint contribution of tibial pixels and magnification rather than of tibial pixels alone (Section 4.9).

The residual 0.257 need not be attributed to extra-tibial anatomy. Zeroing the mask leaves its silhouette, so the outline of the tibia—including the plateau contour, drawn by an annotator who knew the diagnosis—survives the ablation, together with the position and extent of the mask within the frame (Figure S2).

We therefore fitted a logistic regression on the mask silhouette alone, using no radiograph pixel at all and the same partitions. On a 16 × 16 grid it reached 0.213 ± 0.034, above chance (*t* = 3.06, df = 4, *p* = 0.038) and above its own label-permuted null of 0.144 ± 0.032 by +0.069 (95% CI +0.022 to +0.117, *t* = 4.07, df = 4, *p* = 0.015). It is not distinguishable from the tibia-erased network, which exceeds it by +0.043 (95% CI −0.004 to +0.091, *t* = 2.53, df = 4, *p* = 0.065; Figure 3A). On a 48 × 48 grid the same model fell to 0.159 ± 0.019, at chance, which we attribute to fitting 2304 features to roughly 220 training images; neither grid size was specified in advance and both are reported. The attribution below therefore rests on the 16 × 16 grid alone.

Read on its own, that ablation invites the conclusion that the model is not using the target bone. We tested the conclusion by running the complement. Setting every pixel outside the tibial mask to zero produces an input that is the exact complement of the erased one, prepared through the same resize and padding, so the two share a field of view and between them partition the radiograph. That model reached 0.3675 ± 0.0119, exceeding the erased model by +0.111 (95% CI +0.088 to +0.133, *t* = 13.5, df = 4, *p* = 0.0002; Figure 3A) on identical folds. It also matched the mask-ROI crop that carries the surrounding structures, exceeding it by +0.023 (95% CI −0.011 to +0.057, *t* = 1.88, df = 4, *p* = 0.133). Those two models were fitted by different code, so we reran the erased input through the implementation used for its complement and added the whole radiograph, giving three inputs that share one frame and one code path. The erased input reproduced itself (0.266 ± 0.032 against 0.257 ± 0.025, *p* = 0.699), and against that value the tibia alone leads by +0.102 (95% CI +0.054 to +0.149, *p* = 0.004). The whole radiograph, which contains both, reached 0.297 ± 0.032: below the tibia alone by 0.071 (95% CI +0.031 to +0.111, *t* = 4.90, df = 4, *p* = 0.008) and not distinguishable from the erasure (+0.031, 95% CI −0.046 to +0.108, *t* = 1.11, df = 4, *p* = 0.330). On one frame the ordering is therefore the tibia alone, then the whole radiograph, then the erasure (Figure 3A, Table 2). Weighting every patient equally in the loss moved the mask-ROI result by −0.007 (0.338 ± 0.025 against 0.345 ± 0.022; 95% CI −0.039 to +0.026, *t* = −0.58, df = 4, *p* = 0.594). On the Schatzker VI recast the same input reached 0.838 ± 0.036 against 0.685 ± 0.037 erased, a difference of +0.153 (95% CI +0.118 to +0.187, *p* = 0.0002), again above the 0.793 of the full crop. This configuration was run with the GPU reimplementation described in the Materials and Methods, which reproduces the original protocol to within +0.0001 on the mask-ROI input.

About half of what survives the erasure is reproduced by the annotator’s outline of the bone rather than by the anatomy left in the frame. As elsewhere in this study the five-seed comparisons are underpowered, so the equivalence with the mask-ROI crop is bounded rather than established. What the ordering of the three inputs implies for the location of the signal is taken up in Section 4.4.

The centre crop, which uses no mask, reached 0.260 ± 0.031, a paired difference from the tibia-erased input of +0.003 (95% CI −0.020 to +0.026, *p* = 0.747). It was intended to isolate the contribution of the mask but changes the field of view at the same time, capturing a median 0.447 of the tibial bounding box and none of it in 22 of 419 images, so we draw no inference from it about annotation leakage.

### 3.6. Recall Is Graded and Concentrated in Schatzker VI

Per-class recall pooled over five seeds is uneven (Figure 3B, Table S5). Each of the 128 patients contributes five evaluations, so the denominators below are five times the number of patients in each class and the intervals quoted are patient-level cluster bootstrap intervals. Recall runs from 0.72 for Schatzker VI (119/165, 95% CI 0.60 to 0.83) and 0.57 for type I (74/130, 0.45 to 0.68) down to 0.11 for type V (7/65, 0.05 to 0.18) and 0.04 for type IV (2/50, 0.00 to 0.10); types II and III fall between, at 0.43 (0.31 to 0.56) and 0.20 (0.07 to 0.37). The two smallest classes are described better by their per-patient outcomes than by either interval: eight of the ten type IV patients were never classified correctly under any of the five seeds and the remaining two were correct once each, and seven of the thirteen type V patients were never correct.

The aggregate six-class result is carried mainly by Schatzker VI. Recast from the six-class confusion matrix as Schatzker VI against all other fracture types, balanced accuracy is 0.793 ± 0.021 (95% CI 0.729–0.855); no such classifier was trained or thresholded, and the mask ROI supplies the localisation. Averaging recall over the five classes other than VI gives 0.269 ± 0.031, against 0.148 ± 0.025 for the same quantity under permuted labels; predictions remain six-way throughout, so this is not a five-class problem that anyone solved.

Both figures fall when the tibial pixels are erased, but neither falls to chance. The VI-versus-rest recast drops to 0.685 ± 0.037 (95% CI 0.614–0.753) against a permuted null of 0.502 ± 0.037, so 63% of the margin above chance does not require the pixels of the target bone; the five-class average drops to 0.196 ± 0.035. The annotator’s mask silhouette does not account for this: fitted to the same recast it reaches 0.545 ± 0.042, a margin of 0.045 against the 0.293 of the intact model. What survives erasing the tibia in the Schatzker VI signal is therefore reproduced by neither the annotation nor the frame (Section 4.10).

Errors are structured rather than diffuse (Figure 3C). Of Schatzker IV patient evaluations, 40% (20/50 over five seeds) were assigned to type I, 12% (6/50) to type III and 2% (1/50) to type II, so 54% went to a lateral-plateau class; 43% of Schatzker V evaluations (28/65) were assigned to type VI. Schatzker IV is the medial-condyle pattern and types I to III are lateral; the AP appearance of types IV and V is shown in Figure 3D.

The augmentation audit identified a candidate explanation for the direction of the type IV errors that lies in the training pipeline rather than in the radiograph. Horizontal flipping was applied with probability 0.5 to every configuration and the mask ROI excludes the laterality marker, so the network was trained to be invariant to the mirror image of its input. The reasoning that leads from that invariance to a prediction about type IV is set out in Section 4.6.

We tested that explanation by retraining without the flip, holding the partitions, seeds, epochs, optimiser, loss and remaining augmentations fixed. Because this run used a reimplementation that performs augmentation on the GPU, we also reran the flip-on configuration through the same code to give a single-variable contrast; it reproduced the original protocol to three decimal places (0.3447 ± 0.0215 against 0.3446 ± 0.0302, difference +0.0001, 95% CI −0.058 to +0.058, *p* = 0.996), and all three runs share identical folds.

Removing the flip did not recover type IV. Recall moved from 0.060 (3/50) to 0.100 (5/50), a paired difference of +0.040 (95% CI −0.102 to +0.182, *p* = 0.477), leaving it below the chance level of 0.167 in both configurations. The direction-specific prediction failed: the share of type IV evaluations assigned to a lateral class was 54% (27/50) with the flip and 56% (28/50) without it. Six-class balanced accuracy did not improve either, falling from 0.3447 ± 0.0215 to 0.3094 ± 0.0282 (−0.035, 95% CI −0.087 to +0.017, *p* = 0.132), and type V recall fell from 0.108 to 0.046. Schatzker VI against the rest was unchanged, 0.763 ± 0.022 against 0.758 ± 0.029 (*p* = 0.771).

The mirror invariance is therefore real as a property of the trained network but is not the explanation for the type IV collapse, and we withdraw it as one. A further 42% of type IV evaluations (21/50) went to types V and VI, which the mirror operation never explained, and the reverse flow is weak, with 6% of type I evaluations (8/130) assigned to type IV. What remains unaccounted for, and what would be needed to account for it, is taken up in Section 4.6.

### 3.7. Permutation, Convergence and Attribution Controls

Label permutation returned both tasks to chance: 0.491 against 0.500 for the binary task and 0.172 against 0.167 for six classes (Figure 2B and Figure 3A). The partitioning and evaluation pipeline therefore does not leak labels. A seven-class variant that retains NTPF as a class alongside Schatzker I to VI reached 0.352 ± 0.015 under the same protocol (Table S3, Figure S3); it is not discussed further, because the CT confound applies to it as it does to the binary task.

**Figure 4:**
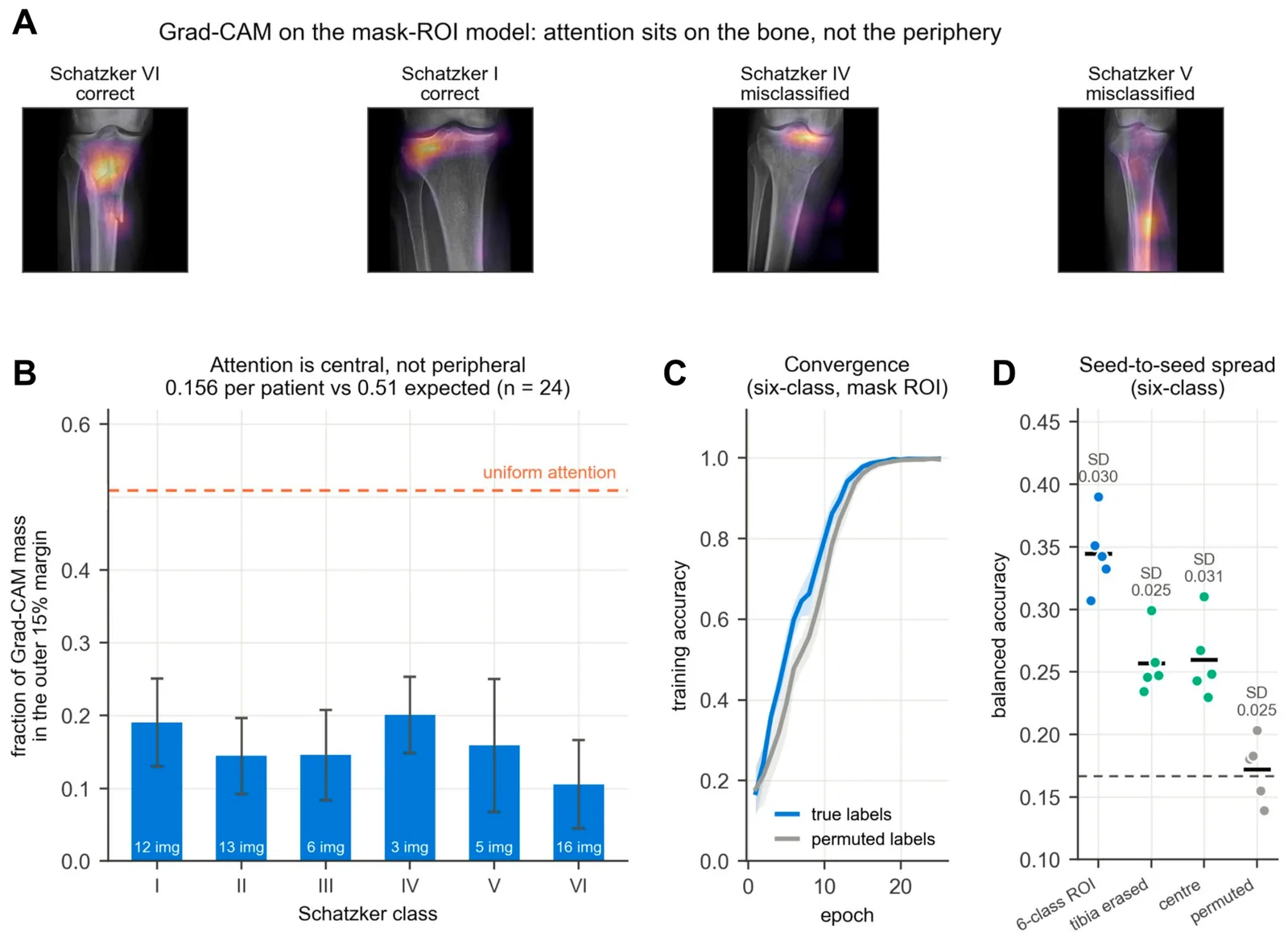
Attribution and control experiments. (**A**) Grad-CAM maps from the last residual block of the mask-ROI six-class model, overlaid on four test cases from fold 1 of seed 0; this analysis is exploratory: one correctly classified Schatzker VI, one correctly classified Schatzker I, one misclassified Schatzker IV and one misclassified Schatzker V. Warm colours indicate higher attribution. (**B**) Fraction of Grad-CAM mass falling in the outer 15% margin of the input, by class, for 55 images from 24 patients; the number of images per class is printed inside each bar, and the value in the title is the mean over the 24 patient means. The dashed line at 0.509 is the value a spatially uniform map would produce, since that margin occupies 0.509 of the image area. (**C**) Training accuracy against epoch for the six-class mask-ROI model trained on true labels and on permuted labels; the line is the mean and the band the standard deviation over five seeds. (**D**) Balanced accuracy of each of the five seeds for four six-class configurations, with the standard deviation across seeds printed above.

Saliency concentrates on the proximal tibia in the correctly classified cases; in the misclassified Schatzker V case it sits on the diaphysis instead (Figure 4A). Grad-CAM mass in the outer 15% margin of the mask-ROI input was 0.156 ± 0.046 per patient, against 0.509 expected under uniform attention (*n* = 24 patients, *t* = −37.3, df = 23, *p* = 4.5 × 10^−22^; Figure 4B). Because the mask ROI is centred on the tibia by construction, this rules out a border artefact but does not by itself show that the model reads fracture morphology, and it says nothing about where the tibia-erased model finds its signal, which the ablation addresses instead. These maps come from one fold of one seed and 24 patients, and the analysis is exploratory.

Both true and permuted labels were memorised, so the models were not under-trained: final training accuracy was 0.997 with true labels and 0.996 with permuted labels on the binary task, and 0.998 and 0.996 on the six-class task (Figure 4C plots the six-class curves; per-seed values for both tasks are in Table S3). This does not establish that the fixed configuration is near the achievable ceiling (Section 4.9).

Seed-to-seed standard deviations were 0.025 to 0.031 across the four six-class configurations shown (Figure 4D, Table S3), small relative to the differences under discussion. Fold-level values for every convolutional configuration and seed are given in Figure S3; fold-level spread is wide, which is why inference is drawn at the seed level.

**Figure 5:**
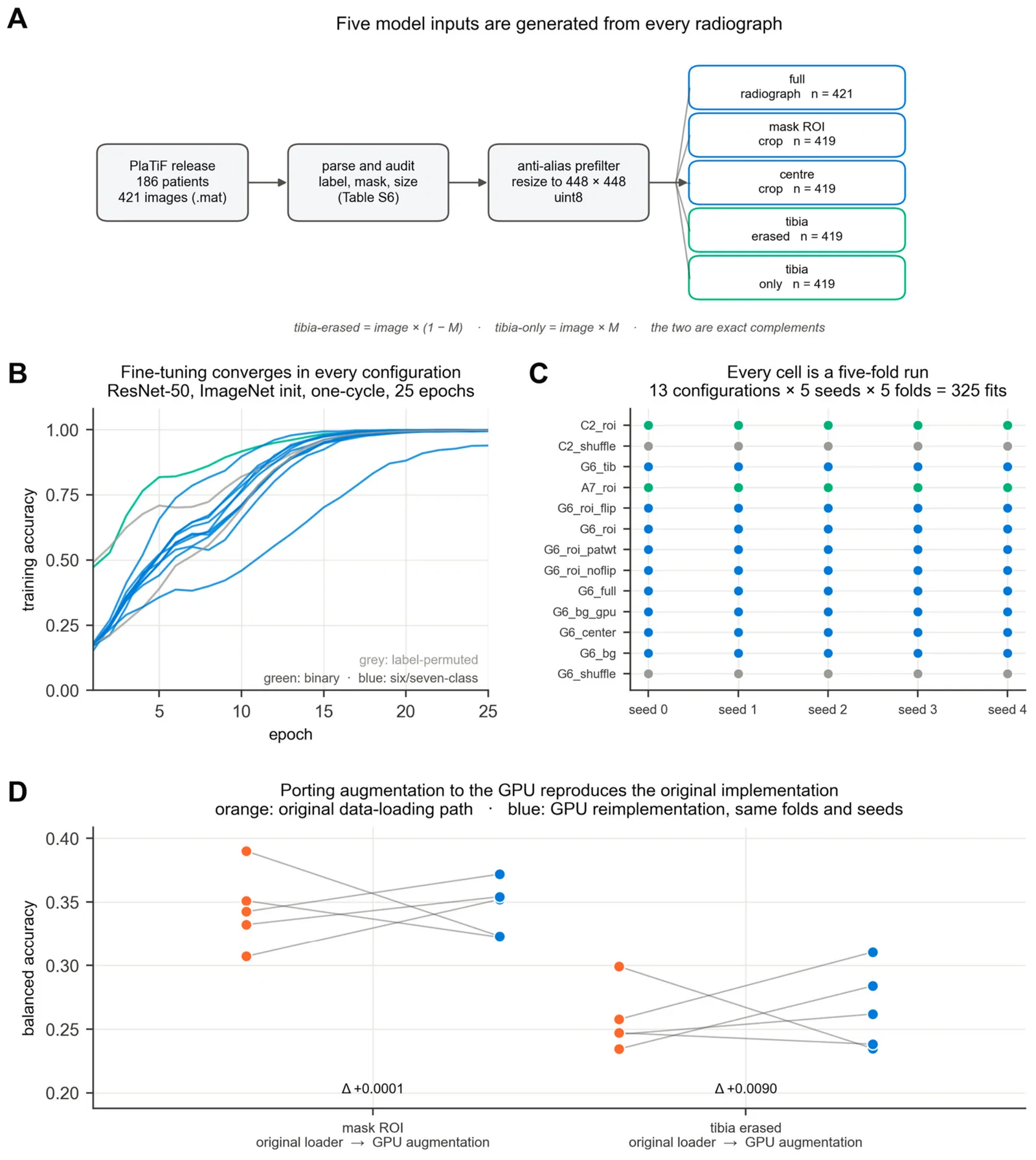
The preprocessing pipeline, the fine-tuning protocol and the experiment grid. (**A**) Every radiograph is parsed from the released MATLAB file, audited, prefiltered against aliasing and resized to 448 × 448, then rendered as five inputs; *n* is the number of images for which each input could be produced, and the tibia-erased and tibia-only inputs are exact complements. (**B**) Training accuracy against epoch, averaged over the twenty-five runs of each configuration; grey lines are the label-permuted configurations, green the binary task and blue the six- and seven-class tasks. (**C**) The grid actually run: one marker per configuration and seed, each standing for a five-fold cross-validation. (**D**) The augmentation path was reimplemented on the GPU when host-CPU contention made the data loader the bottleneck; the two groups compare the original path with the reimplementation on identical folds and seeds, with lines joining the same seed.

Final training accuracy reached at least 0.94 in every configuration and 0.99 or above in twelve of the thirteen, and the complete grid actually run, 13 configurations by five seeds by five folds and so 325 fine-tuning runs, is shown with the preprocessing pipeline and the implementation check in Figure 5. We detected no association between per-class recall and published human inter-observer agreement for Schatzker classification from an AP radiograph alone [3] (Spearman ρ = 0.26, *p* = 0.62, *n* = 6 classes; Figure S4). With six points the comparison has little power. We report it as a null result, not as evidence that model and human difficulty are unrelated.

## 4. Discussion

### 4.1. Principal Findings

Four numbers from this benchmark look like measurements of what a radiograph shows, and not one of them means what it appears to mean. Distinguishing fracture from no classifiable fracture gives 0.833, and a model handed no pixel content gives 0.814 on the same folds. Six-class Schatzker typing gives 0.345, half of which survives erasing the pixels of the target bone, though the tibia on its own turns out to carry more than everything around it. Recast as Schatzker VI against the rest it gives 0.793, of which 63% survives the same erasure. Type IV, the one distinction an AP film shows plainly, is recovered at 0.04, below chance, and the augmentation we built was blind to it by construction, though removing that blindness did not bring the class back. Each of these was exposed by a control that costs minutes, and on this dataset all four returned a finding.

### 4.2. The Four Controls and What Each One Catches

The four are not an arbitrary list. A benchmark score is produced by a chain, and each control cuts the chain at one link: the archive that supplied the cases, the anatomy the label names, the annotation shipped alongside it, and the recipe used to fit the model. Each is a different candidate author of the same number, and on this dataset each of them returned a finding. Two returned findings against readings we had already committed to, which is the strongest argument we can make for running them. Section 2.6 states the four in the notation that makes them transferable; the sections below take each in turn.

### 4.3. Confounding by Indication Disqualifies One Task

The first control removes one task from consideration as a measure of image interpretation. Distinguishing patients with a Schatzker-classifiable fracture from those without one looks easy, but a model given no pixel content matches the network on the same folds, because a coronal CT accompanies almost every fracture patient and fewer than half of the others, so that label is very nearly a record of which patients were investigated for a plateau fracture (Section 3.2, Table S7). This is confounding by indication, a property of how a clinical archive is assembled rather than a flaw peculiar to this release. The network cannot exploit it directly, since the CT variable is not among its inputs; what it does is make the task easier than the radiograph alone makes it. We restricted the rest of the analysis to the 128 fracture patients, where coronal CT is close to constant, and we report the binary result as a caution rather than as a finding.

With that task set aside, the six-class comparison is informative: on identical folds the image model leads a baseline given no pixel content by +0.168, against a chance level of 0.167 (Section 3.4, Table 2). Recall is graded rather than uniform and the errors are directed, type IV into the lateral classes and type V into type VI.

### 4.4. Ablation Must Be Reported in Pairs

The second control is the one that would have misled us, and it is the reason we now recommend running ablations in pairs. Erasing the tibial pixels leaves half the six-class margin above chance standing, and on the Schatzker VI recast it costs only about a third. Reported alone, that is naturally read as the model working largely without the bone it is classifying. The complement says otherwise: the tibia with everything else removed exceeds its own erasure by +0.111, and it is not below the crop that contains the whole knee (Section 3.5, Figure 3A, Table 2). The two inputs partition the radiograph and share a field of view, so this is a like-for-like contrast in a way that neither is against the mask ROI.

What the pair establishes is that the signal is concentrated in the target bone, and the third input settles it: on one frame and one code path a network given the whole radiograph does no better than a network given everything except the tibia (Section 3.5). The femur, the fibula and the soft-tissue envelope stay in the erased image and a severe injury marks all three, which is a plausible source for the residual; but that residual is not a second copy of the same information, because the model holding both copies does not exceed the one holding the surround alone. An ablation bounds what a model can do without something; it does not bound what the model does with it, and only the complement speaks to that.

### 4.5. Leakage from a Distributed Expert Segmentation

The third control asks whether an expert annotation supplies what the ablation leaves behind, and the answer differs by task. Zeroing the mask leaves its silhouette, the outline of the tibia drawn by someone who knew the diagnosis, positioned and scaled by how the limb was framed. A logistic regression given nothing but that binary shape reaches above chance and the tibia-erased network does not separate from it, so measured above chance the outline accounts for about half of the six-class residual, on a comparison that fails to separate the two rather than one that equates them (Section 3.5). On the Schatzker VI recast the same regression accounts for almost none of it.

This is a general hazard for datasets that ship expert segmentations alongside labels. A mask is usually treated as neutral localisation, but it is an expert annotation of the same anatomy the label describes, and any ablation that preserves its geometry keeps an information channel open. Reporting what a model can do from the mask alone costs one logistic regression and settles the question.

### 4.6. Testing and Rejecting One Explanation for the Failure of Schatzker IV and V

Returning to the two directed confusions, type IV into the lateral classes and type V into type VI, they have different explanations, and only one of them is about the radiograph. Types V and VI differ in how far the fracture runs into the diaphysis, which the AP view resolves poorly once the metaphysis is superimposed on the proximal shaft, and the mask ROI compounds it because the crop ends where the annotator’s mask ends. Depth of depression and posterior-column involvement are likewise unavailable in a single projection, which is why Schatzker himself, with Kfuri, moved the classification onto CT [4].

Type IV is different. Medial versus lateral is the one distinction an AP film shows plainly, and human observers reading that film agree on type IV as well as on type II; our model nonetheless assigns 54% of type IV evaluations to a lateral class. The fourth control looked for a reason in the training pipeline rather than in the projection and found one: the network had been trained to treat a fracture and its mirror image as the same input, which on the reading that it takes laterality from the side of the frame predicts exactly the misassignment we observe. It was testable in half an hour, so we tested it, and it did not survive (Section 3.6).

We report this because it is the point of running the control at all. Had we stopped at the coincidence, the manuscript would have carried a mechanical explanation that is wrong, stated with the confidence that a matching error pattern invites. What remains is a collapse we cannot attribute, and two explanations remain that this design does not separate: the AP projection may not carry the medial–lateral distinction once the condyles are superimposed, or the classes may be too small to learn, at 10 patients for type IV and 13 for type V. The failure is not confined to a few hard cases within them: eight of the ten type IV patients were never classified correctly under any of the five seeds, and seven of the thirteen type V patients were never correct. The direction of the errors fits the projection better than scarcity, but the flip result shows how far a matching error direction is from a demonstrated mechanism, and across the six types we detected no association between model recall and published human agreement (Figure S4). Every statement about types IV and V in this paper is therefore bounded by the size of those classes, and Section 4.10 states what would be needed to separate the two explanations.

### 4.7. Comparison with the Existing Literature

The one closely comparable published study points the same way overall. Working with two radiographic projections rather than one, and with a larger multicentre cohort, van der Gaast and colleagues report 34.6% unweighted accuracy for Schatzker typing on their test split [6]. That number is not directly comparable with ours: it is unweighted rather than balanced, their cohort of 753 patients includes 385 knees without a fracture, and their evaluation split differs from ours. The comparable quantity on our side is unweighted accuracy, which is 0.448. What the two studies share is a direction rather than a value—supervised Schatzker typing from plain radiographs remains far from the reliability with which the classification is used—and neither is designed to test whether more data would move it.

### 4.8. Implications for Practice and Deployment

#### 4.8.1. What This Dataset Shows

Two numbers describe what this release supports, and both are internal cross-validated estimates within 186 patients of a single centre rather than generalisation estimates. The figure available for a coarse severity judgement, 0.793, is recast from the six-class confusion matrix under oracle localisation; no Schatzker VI versus rest classifier was trained or thresholded, so it is not a measured performance and a classifier fitted to that objective could fall on either side of it. It is optimistic for a second reason as well, the oracle localisation it assumes. The presence or absence of a coronal CT series carried a balanced accuracy of 0.785 on its own, which is a property of the archive and not of the radiograph. Neither number was obtained in a configuration that could be deployed.

#### 4.8.2. What Would Follow for Practice

Three implications follow, and none of them rests on the values just quoted as a level of performance. First, a model intended to type tibial plateau fractures should be given the coronal information the classification is defined on, either as CT or, at minimum, as a second projection; the alternative is to accept that the AP view supports a coarse severity judgement rather than a six-way type. Second, public releases should distribute the pathway variables alongside the images, because here the presence or absence of a CT series was recoverable from the archive and, had it not been, the binary benchmark would have looked like a solved problem. Third, every accuracy we report on mask-ROI inputs assumes a localisation the annotators supplied. We trained no segmentation model, and an end-to-end system would have to earn that localisation before it could claim these numbers.

A clinician or department evaluating a tool of this kind is usually shown a single accuracy, and the results here indicate how little that number constrains what the tool is doing. On this dataset the headline figure for separating fractured from non-classifiable knees, 0.833, was matched by a model that saw no pixel at all, because the label tracked whether the patient had been sent for a coronal CT. A tool validated on such an archive would appear accurate in retrospective testing and would fail in exactly the situation it is bought for, the patient whose imaging pathway has not yet been decided. Nothing about that failure is visible in the reported metric.

On the strength of one dataset we do not propose these as a standard, but they are cheap enough to be asked for, and a reader evaluating a tool or a preprint can reasonably ask which of them were run: a comparator fitted to non-image variables on the same partitions, an ablation of the target anatomy together with its complement, a model given the annotation alone where segmentations are used, and a statement of which augmentations were applied. None requires access to the model weights. Whether a control of this kind reliably detects a given failure is itself untested, and Section 4.10 states what would be needed to calibrate them.

### 4.9. Limitations

Four limitations bound what this study can claim. First, the cohort is 186 patients from a single centre and there is no external validation set, so no value reported here is a generalisation estimate. Second, the six-class task is small and unevenly filled, and types IV and V contain 10 and 13 patients. Third, every accuracy on the mask-ROI input assumes an expert segmentation supplied at inference, which no deployed system would have. Fourth, the attribution analysis is exploratory. The paragraphs below take these four in turn and then state the narrower caveats that attach to individual comparisons.

The cohort is 186 patients from one hospital and there is no external validation set, so the absolute values reported here should not be read as generalisable performance. What we rest on is nevertheless not a performance estimate: it is a set of comparisons between models trained and evaluated on identical partitions, and the ordering of those comparisons is what the argument uses.

We fixed one architecture and one training protocol in advance and performed no hyperparameter search. A better-regularised or better-tuned model might separate from the non-anatomical baseline on the binary task, and the negative binary result is specific to the configuration reported here. What does not depend on the configuration is the confound itself: a single rule on CT availability reaches 0.785 balanced accuracy with no image at all, and that number is a property of the release.

The binary comparison is underpowered in two distinct ways: its paired test has four degrees of freedom, and its interval describes training randomness and partition redrawing within a fixed cohort rather than uncertainty about which patients the cohort contains. We therefore report it as a failure to distinguish rather than as evidence of equivalence. The six-class comparison, where the separation is several times the seed-to-seed spread, does not depend on this resolution.

The mask-ROI input uses a segmentation produced by the dataset’s annotators and supplied at inference time. It is an oracle crop and not a deployable configuration: it sets an upper bound on what an automatic pipeline could achieve, which is the appropriate direction for the negative conclusions drawn here, but it means the reported six-class accuracy is optimistic relative to a fully automatic system. We cannot say by how much. No segmentation model was trained in this study, so the cost of replacing the expert mask with an automatic one is not measured here, and it would have to be measured before any of these accuracies could be claimed for an end-to-end system. This dependence is the single largest limit on the clinical applicability of the configuration we benchmarked, and it applies to every mask-ROI result in the paper, including the 0.793 recast and the per-class recalls.

The attribution analysis is exploratory. Grad-CAM maps were computed at one layer of one model, for a single fold of a single seed, and for 55 images from 24 patients; no map was computed for the tibia-erased model. The margin-mass statistic rules out a border artefact, but we do not use the maps as evidence about which features the network reads, and no conclusion of this study rests on them.

Two of the input comparisons change the field of view as well as the content, and we do not separate the two effects: the centre crop captures a median 0.447 of the tibial bounding box, so we draw no conclusion from it, and the +0.088 between the mask ROI and the tibia-erased input bounds the joint contribution of tibial pixels and magnification. The tibia-only input was added for that reason, since it shares a field of view with the erased input exactly. Neither point affects the comparisons the paper rests on, which are between models on identical partitions.

The tibia-only configuration was run with the GPU reimplementation of the training loop rather than the original data-loading path. The two implementations agree to +0.0001 on the mask-ROI input (*p* = 0.996), the erased input reproduced its original value through the reimplementation (*p* = 0.699), and all configurations share identical folds, so the contrast between the erasure and its complement no longer straddles two code paths.

Fixing a single comparator in advance protects against reporting the weakest of many, but it does not make the one chosen the strongest available, and on these data it was not: the margin we report is +0.168 against the comparator we fixed and +0.093 against the best feature block we found afterwards (Table S7). The ordering of the comparison is unchanged either way. The mask-silhouette control was added after the main experiments and its grid size was not prespecified, so the attribution we draw holds at one of the two resolutions we examined and not at the other. Two further caveats attach to it: the comparison it supports is a null, and with five seeds a null is weak evidence; and the silhouette model is a linear classifier on a coarse binary image, so its value is a lower bound on what the outline carries, and a non-difference between models of unequal capacity does not establish that the two are reading the same thing. The control is enough to rule out an extra-tibial-anatomy reading of the residual, which is how we use it, but not enough to establish that the silhouette accounts for all of it.

The competing explanations for the collapse of types IV and V, the limits of the projection and the size of the classes, are set out in Section 4.6; this design separates neither, and we draw no stronger conclusion than that the collapse is real and its cause is open. Our own first explanation, the mirror invariance introduced by horizontal flipping, was tested and did not hold, so we no longer offer it. Separating the two that remain would need either a cohort with enough type IV patients to learn the class or paired CT for the same patients, neither of which this release provides.

Per-class recall is pooled over five seeds, so each patient contributes five evaluations and those evaluations are correlated. Figure 3B therefore reports patient-level cluster bootstrap intervals, in which the resampling unit is the patient and all five evaluations of a drawn patient enter together. Clopper–Pearson intervals over the pooled evaluations, which treat the five as independent, are 1.3 to 1.6 times narrower in types I, II, III and VI; both are tabulated in Table S5. In types IV and V the clustered interval is the narrower of the two, because almost every patient of those classes is misclassified under every seed and the resampled distribution is close to degenerate, so for those two classes the per-patient outcomes given in Section 3.6 describe the failure better than either interval does. The ordering of the classes, which is what the argument uses, is unaffected under either scheme: taken one seed at a time, recall for type IV ranges from 0.00 to 0.10 and for type V from 0.08 to 0.23, against 0.67 to 0.76 for type VI.

Two features of the release remain unexplained: the reported cohort age does not reproduce from the released metadata, and the image-level label disagrees with the metadata spreadsheet for four patients. A third observation, the coronal CT count, is a documentation inconsistency rather than a defect of the data. We used the image-level label throughout, because it is the label attached to the pixels being classified; removing the four affected patients instead gives 0.340 ± 0.037 against 0.345 ± 0.030, so that choice does not carry the result.

### 4.10. Future Directions

Three lines follow directly. First, the failure of Schatzker IV remains unexplained once the augmentation is excluded as its cause; resolving it needs the larger type IV cohort or the paired CT identified in Section 4.6. Second, the residual that survives erasing the tibia in the Schatzker VI signal was reproduced neither by the annotation nor by the framing variables, and locating it would require attribution methods applied to the ablated model rather than to the intact one. Third, the controls themselves should be tested on benchmarks where the answer is known, so that the size of the effect each one detects can be calibrated rather than merely reported.

## 5. Conclusions

A convolutional network separated the six Schatzker types on single anteroposterior radiographs well above chance, given an expert segmentation supplied at inference that no deployed system would have, and four controls, each costing minutes, showed that the number does not mean what it appears to mean. One task in the release was uninterpretable because coronal CT availability tracked the label. Half of the six-class margin survived erasing the pixels of the target bone, yet the complementary input, the tibia alone, exceeded both that erasure and the whole radiograph. The signal is therefore concentrated in the target bone rather than duplicated around it, and the ablation alone would have been read the wrong way. About half of what survived the erasure was matched by a regression on the annotator’s mask with no radiograph pixels at all, on a comparison that failed to separate the two (*p* = 0.065). An audit of the augmentations identified a mirror invariance we had introduced, which removes the side of the frame on which a fracture appears; retraining without it did not recover Schatzker IV, which removed our own pipeline as the explanation and left that failure open.

We do not conclude that radiographic Schatzker classification is hopeless, nor that this dataset is unusable. We conclude that a benchmark accuracy computed on a retrospective clinical archive is not by itself evidence that a model read the anatomy named by the label, that ablations reported in one direction can invert their own meaning, and that controls of this kind are cheap enough to be worth running, although a single dataset cannot establish how generally they are required.

## Figures and Tables

**Table 1:** Cohort and acquisition characteristics of the PlaTiF release.

Characteristic	Value
Patients	186
Radiographs	421
Images per patient, median (range)	2 (1–8)
Age, years, mean ± standard deviation (SD) (range)	43.60 ± 17.85 (15–88)
Reported in the data descriptor	45.88 ± 17.54 (*n* = 186)
Sex, male/female	149/37
Fractured side, R/L/bilateral	96/87/3
Coronal computed tomography (CT) available	150/186 (80.6%)
Schatzker I/II/III/IV/V/VI	27/34/12/11/12/32
Image-level labels used for modelling	26/34/12/10/13/33
NTPF (no Schatzker-classifiable fracture)	58
Image height, px, range	1467–4892
Image width, px, range	920–4892
Aspect ratio (h/w), range	0.75–3.07
Images extending beyond the knee (height > 4000 px or width > 3000 px)	51/421
Label conflicts (.mat vs. metadata)	4 patients
Images with an unusable mask	2/421

**Table 2:** Balanced accuracy of every configuration on identical patient-level partitions.

Task	*n* Patients	Chance	Input/Model	Balanced Accuracy (Mean ± SD over 5 Seeds)	Patient-Cluster Bootstrap 95% CI	Macro-F1 (Mean ± SD)	vs. Prespecified Non-Anatomical Baseline	Note
Binary: NTPF vs. fracture	186	0.500	label-permutation null	0.491 ± 0.048	0.462 to 0.521	0.486 ± 0.055		control
Binary: NTPF vs. fracture	186	0.500	mask geometry only (logistic)	0.564 ± 0.021		0.549 ± 0.020		not prespecified
Binary: NTPF vs. fracture	186	0.500	non-anatomical baseline (logistic)	0.814 ± 0.016		0.816 ± 0.015		prespecified comparator
Binary: NTPF vs. fracture	186	0.500	ResNet-50, mask ROI	0.833 ± 0.028	0.782 to 0.882	0.831 ± 0.024	+0.019 [−0.022, +0.060], *p* = 0.264	not distinguishable
Six-class Schatzker (fracture patients)	128	0.167	label-permutation null	0.172 ± 0.025	0.146 to 0.200	0.171 ± 0.030		control
Six-class Schatzker (fracture patients)	128	0.167	mask geometry only (logistic)	0.138 ± 0.018		0.130 ± 0.017		not prespecified
Six-class Schatzker (fracture patients)	128	0.167	non-anatomical baseline (logistic)	0.177 ± 0.026		0.161 ± 0.022		prespecified; just above chance
Six-class Schatzker (fracture patients)	128	0.167	mask silhouette only, 16 × 16 (logistic)	0.213 ± 0.034		0.210 ± 0.034		no radiograph pixels; permuted null 0.144 ± 0.032
Six-class Schatzker (fracture patients)	128	0.167	ResNet-50, tibia erased	0.257 ± 0.025	0.211 to 0.305	0.246 ± 0.028	+0.080 [+0.022, +0.139], *p* = 0.019	full frame; mask silhouette retained
Six-class Schatzker (fracture patients)	128	0.167	ResNet-50, tibia only	0.367 ± 0.012		0.364 ± 0.013	+0.191 [+0.146, +0.236], *p* = 0.0003	exact complement of the row above, same field of view; +0.111 [+0.088, +0.133], *p* = 0.0002 against it
Six-class Schatzker (fracture patients)	128	0.167	ResNet-50, centre crop	0.260 ± 0.031	0.220 to 0.301	0.252 ± 0.033	+0.083 [+0.020, +0.146], *p* = 0.022	no mask used; field of view differs
Six-class Schatzker (fracture patients)	128	0.167	ResNet-50, mask ROI	0.345 ± 0.030	0.301 to 0.389	0.335 ± 0.031	+0.168 [+0.106, +0.230], *p* = 0.002	primary result; oracle localisation
Six-class Schatzker (fracture patients)	128	0.167	ResNet-50, mask ROI, GPU-augmentation replicate	0.345 ± 0.022		0.338 ± 0.021	+0.168 [+0.131, +0.205], *p* = 0.0002	flip-on control for the row below; reproduces the row above (+0.0001 [−0.058, +0.058], *p* = 0.996)
Six-class Schatzker (fracture patients)	128	0.167	ResNet-50, mask ROI, no horizontal flip	0.309 ± 0.028		0.298 ± 0.027	+0.133 [+0.095, +0.170], *p* = 0.0006	augmentation audit; vs. flip-on replicate −0.035 [−0.087, +0.017], *p* = 0.132
Six-class Schatzker (fracture patients)	128	0.167	ResNet-50, whole radiograph	0.297 ± 0.032		0.286 ± 0.030	+0.120 [+0.054, +0.185], *p* = 0.007	full frame, no crop and no mask; same folds and code path as tibia-only
Six-class Schatzker (fracture patients)	128	0.167	ResNet-50, tibia erased, GPU-augmentation replicate	0.266 ± 0.032		0.257 ± 0.032	+0.089 [+0.062, +0.116], *p* = 0.0008	same code path as tibia-only and whole radiograph
Six-class Schatzker (fracture patients)	128	0.167	ResNet-50, mask ROI, patient-weighted loss	0.338 ± 0.025		0.331 ± 0.025	+0.161 [+0.105, +0.218], *p* = 0.001	each image weighted 1/n images of its patient; sensitivity analysis
Seven-class (all patients)	186	0.143	ResNet-50, mask ROI	0.352 ± 0.015	0.315 to 0.389	0.345 ± 0.014		secondary; CT confound applies
Schatzker VI vs. rest (recast)	128	0.500	label-permutation null	0.502 ± 0.037	0.464 to 0.540			control
Schatzker VI vs. rest (recast)	128	0.500	mask silhouette only, 16 × 16 (logistic)	0.545 ± 0.042				no radiograph pixels
Schatzker VI vs. rest (recast)	128	0.500	ResNet-50, centre crop	0.654 ± 0.034	0.580 to 0.727			no mask used
Schatzker VI vs. rest (recast)	128	0.500	ResNet-50, tibia erased	0.685 ± 0.037	0.614 to 0.753			63% of the margin above chance retained
Schatzker VI vs. rest (recast)	128	0.500	ResNet-50, tibia only	0.838 ± 0.036				complement of the row above; +0.153 [+0.118, +0.187], *p* = 0.0002 against it
Schatzker VI vs. rest (recast)	128	0.500	ResNet-50, mask ROI	0.793 ± 0.021	0.729 to 0.855			recast from the six-class confusion matrix; no classifier trained
								bootstrap intervals resample patients within class, carrying all five of a patient’s evaluations together. Differences in the penultimate column are paired across seeds. The patient-cluster bootstrap of the mask ROI minus tibia-erased difference is +0.088 [+0.043, +0.132]. Intervals for the logistic-regression rows were computed by resampling evaluations and are omitted rather than reported on a different basis. The Schatzker VI versus remaining rows are recast from the six-class confusion matrix; no binary classifier was trained or thresholded, and the mask ROI supplies the localisation.

## Data Availability

The PlaTiF dataset is publicly available from Zenodo (https://doi.org/10.5281/zenodo.18007397) under CC BY 4.0. All processed per-patient and per-image tables generated in this study, together with the per-seed model results and baselines, are provided as Supplementary Tables S1–S7. Analysis code is available at https://github.com/sanghyunn82-creator/platif-controlled-benchmark (accessed on 8 September 2026).

## Supplementary Materials

The following supporting information can be downloaded at: https://www.mdpi.com/article/10.3390/jcm15187075/s1, Figure S1. The two images whose segmentation mask does not match the radiograph. Figure S2. The five inputs shown for one representative patient of each fracture class. Figure S3. Fold-level balanced accuracy for seven of the convolutional configurations. Figure S4. Per-class recall against published human inter-observer agreement. Table S1. Per-patient characteristics of the PlaTiF release (186 rows). Table S2. Per-image characteristics and mask applicability (421 rows). Table S3. Per-seed results for every convolutional configuration (65 rows). Table S4. Non-anatomical and mask-geometry baselines, per seed (75 rows). Table S5. Pooled per-class recall, precision and confusion for the six-class mask-ROI model, with both patient-level cluster bootstrap and Clopper–Pearson intervals. Table S6. Data integrity findings and the action taken for each (12 rows). Table S7. The non-anatomical comparator broken into feature blocks (12 rows).
