# Supplementary material for: Deep Learning for Schatzker Classification on Anteroposterior Radiographs: A Controlled Benchmark and a Transferable Control Protocol
Source: J Clin Med. 2026 Sep 11;15(18):7075. doi: 10.3390/jcm15187075 (PMC13607838; doi:10.3390/jcm15187075)
Supplement: Supplementary file 1 [file jcm-15-07075-s001.zip › jcm-4546801-supplementary.pdf]

Supplementary Materials

Deep Learning for Schatzker Classification on Anteroposterior Radiographs: A Controlled Benchmark and a Transferable Control Protocol

Sang Hyun Na 1, So Hyun Ahn 2,3,*

1 School of Engineering and Computing, Rice University, 6100 Main St, Houston, TX 77005, United States; sn73@rice.edu
2 Ewha Medical Research Institute, School of Medicine, Ewha Womans University, Seoul, Republic of Korea
3 Ewha Medical Artificial Intelligence Center, School of Medicine, Ewha Womans University, Seoul, Republic of Korea

* Correspondence: mpsohyun@ewha.ac.kr

Contents

Figure S1. The two images whose segmentation mask does not match the radiograph.
Figure S2. The five inputs shown for one representative patient of each fracture class.
Figure S3. Fold-level balanced accuracy for seven of the convolutional configurations.
Figure S4. Per-class recall against published human inter-observer agreement.
Table S1. Per-patient characteristics of the PlaTiF release (186 rows).
Table S2. Per-image characteristics and mask applicability (421 rows).
Table S3. Per-seed results for every convolutional configuration (65 rows).
Table S4. Non-anatomical and mask-geometry baselines, per seed (75 rows).
Table S5. Pooled per-class recall, precision and confusion for the six-class mask-ROI model, with both patient-level cluster bootstrap and Clopper–Pearson intervals.
Table S6. Data integrity findings and the action taken for each (12 rows).
Table S7. The non-anatomical comparator broken into feature blocks (12 rows).

Tables S1, S2, S3, S4 and S7 are supplied as separate machine-readable files (CSV and a single XLSX workbook) because of their length; Tables S5 and S6 are reproduced in full below.

Supplementary Figures

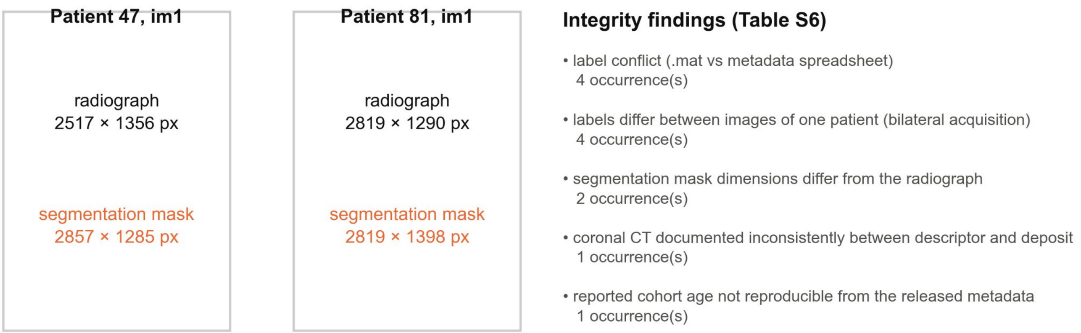

Figure S1. The two images whose segmentation mask does not match the radiograph.

Radiograph and mask dimensions for patient 47 image im1 and patient 81 image im1, with a summary of the integrity findings enumerated in Table S6.

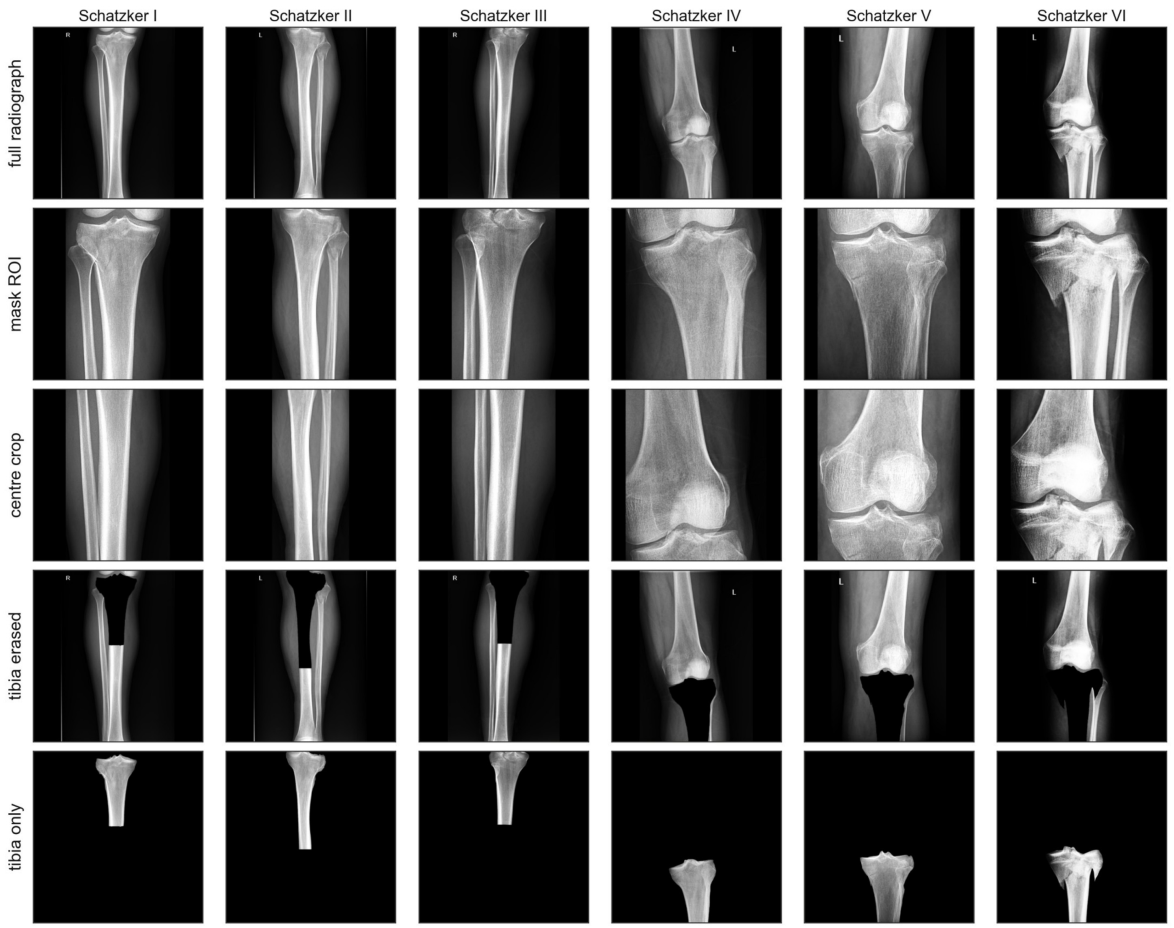

The centre crop uses no mask; across the 419 images with an applicable mask it captures a median 0.447 of the tibial bounding box and none of it in 22 images (Table S2). Erasing the tibia leaves the femur, fibula, joint space and soft tissue intact, and the erased region retains the outline of the annotator's mask. The tibia-only input is the exact co

Figure S2. The five inputs shown for one representative patient of each fracture class.

Rows are the full radiograph, the mask ROI, the centre crop, the tibia-erased input and the tibia-only input; columns are Schatzker I to VI. The centre crop uses no mask. The tibia-erased and tibia-only rows are exact complements of one another: each pixel is retained in one and zeroed in the other, and the two share a field of view.

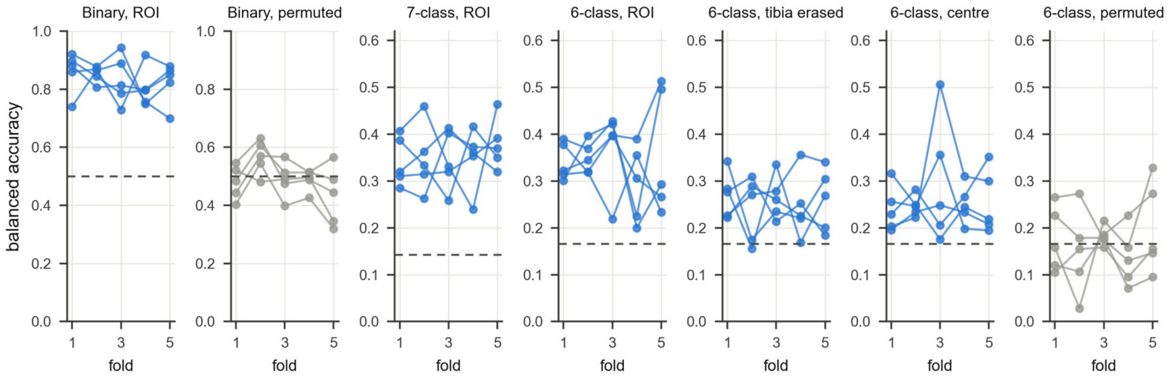

Figure S3. Fold-level balanced accuracy for seven of the convolutional configurations.

Each panel is one configuration; each line is one seed across the five cross-validation folds. The seven panels are the configurations available when the figure was drawn: binary mask-ROI and its label-permuted null, seven-class mask ROI, and six-class mask ROI, tibia-erased, centre crop and label-permuted. The configurations added later, tibia-only, whole-radiograph, the flip-on and flip-off pair and the patient-weighted replicate, are not drawn here; their per-seed and per-fold values are in Table S3. The dashed line is chance for that task. Note the differing vertical scale between the binary and the multi-class panels.

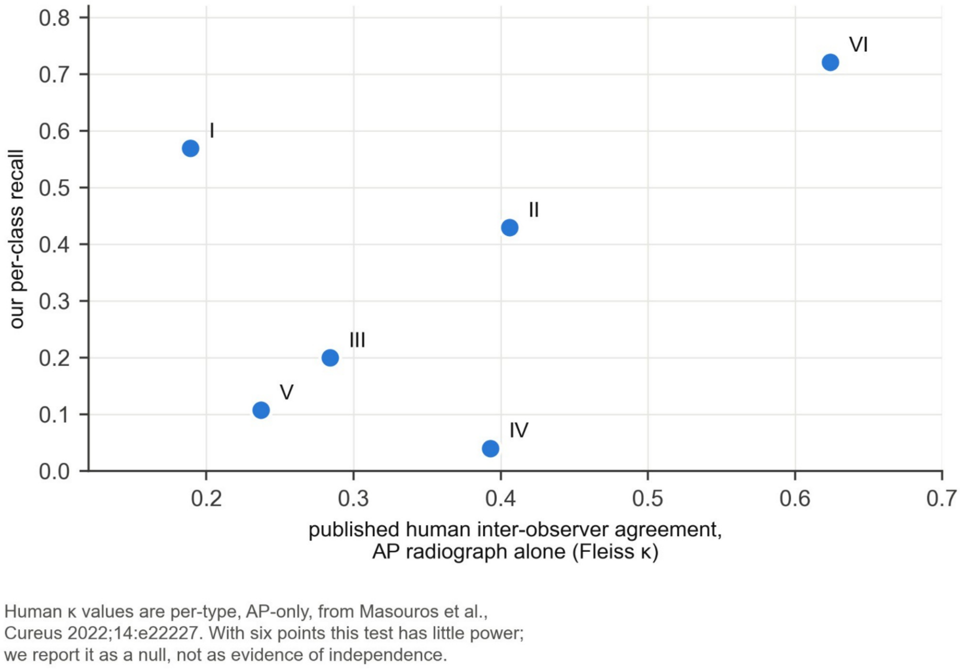

Figure S4. Per-class recall against published human inter-observer agreement.

Model recall for each Schatzker type against the per-type Fleiss kappa reported for classification from an anteroposterior radiograph alone by Masouros et al. [3], in which 12 observers rated 25 cases. Each point is one Schatzker type; higher values on either axis mean better agreement or better recall.

Supplementary Tables

Table S1. Per-patient characteristics of the PlaTiF release (186 rows).

One row per patient. Columns give the patient identifier, the Schatzker label recorded in the metadata spreadsheet, the image-level labels found in the .mat files, whether those labels are constant within the patient, whether the two sources conflict, the number of radiographs available, whether a coronal CT section accompanies the patient, age and sex. The label columns are the source of the four label conflicts and the four bilateral acquisitions enumerated in Table S6, and the coronal CT column is the variable used in Figure 1D and in the confounding analysis.

Supplied as a separate machine-readable file (Table_S1.csv, and as a sheet in Supplementary_Tables.xlsx).

Table S2. Per-image characteristics and mask applicability (421 rows).

One row per radiograph. Columns give the image key, the patient it belongs to, the image index within the patient, height and width in pixels, the aspect ratio, the image-level Schatzker label, whether the distributed segmentation mask can be applied to that image, and the fraction of the frame occupied by the mask. The mask_applicable column identifies the two images whose mask dimensions differ from the radiograph (Figure S1), which are excluded from every mask-dependent input; the height and width columns define the whole-leg criterion used in Figure 1C and Table 1.

Supplied as a separate machine-readable file (Table_S2.csv, and as a sheet in Supplementary_Tables.xlsx).

Table S3. Per-seed results for every convolutional configuration (65 rows).

One row per configuration and seed, for 13 configurations and five seeds each. Columns give the configuration name, seed, architecture, preprocessing version, label source, number of patients, the chance level for that task, balanced accuracy, macro F1, plain accuracy, the final training accuracy, and the five fold-level balanced accuracies. Every value reported in Table 2 and in Figures 2, 3A and 4D is computed from this table. The configurations are: C2_roi and C2_shuffle (binary task and its label-permuted null), A7_roi (seven-class secondary analysis), G6_roi, G6_center, G6_bg and G6_shuffle (six-class mask ROI, centre crop, tibia-erased and permuted null), G6_tib (tibia-only, the complement of G6_bg), and G6_roi_flip and G6_roi_noflip (the flip-on control and the flip-off configuration of the augmentation audit), and G6_full, G6_bg_gpu and G6_roi_patwt (the whole radiograph, the tibia-erased input refitted through the same implementation as G6_tib and G6_full, and the patient-weighted replicate of G6_roi). Every configuration other than C2_roi, C2_shuffle, A7_roi, G6_roi, G6_center, G6_bg and G6_shuffle was run with the GPU reimplementation described in Section 2.4.

Supplied as a separate machine-readable file (Table_S3.csv, and as a sheet in Supplementary_Tables.xlsx).

Table S4. Non-anatomical and mask-geometry baselines, per seed (75 rows).

One row per task, feature set, model and seed. Columns give the task, the feature set (the non-anatomical baseline, stored under the identifier non-imaging metadata; mask geometry; or both), the model (logistic regression or random forest), whether that combination is the prespecified primary comparator, the seed, and the balanced accuracy. The rows flagged as prespecified are the baselines against which every image model is compared in the main text; the remaining rows are reported for completeness and were not prespecified.

Supplied as a separate machine-readable file (Table_S4.csv, and as a sheet in Supplementary_Tables.xlsx).

Table S5. Pooled per-class recall, precision and confusion for the six-class mask-ROI model, with both patient-level cluster bootstrap and Clopper–Pearson intervals.

One row per Schatzker type, pooled over five seeds so that each of the 128 patients contributes five evaluations. Columns give the class, the number of patient evaluations, the number correct, recall with its 95% patient-level cluster bootstrap interval (20,000 resamples, the patient as the resampling unit) and its 95% Clopper–Pearson interval, the ratio of the two interval widths, the number of patients in the class, the number of those patients never classified correctly under any seed, precision, and the full row of the confusion matrix as counts predicted into each class. This table is the numerical form of Figure 3B and 3C, and it is the source of the directional error rates quoted in Section 3.6 (for example, 27 of 50 Schatzker IV evaluations assigned to a lateral-plateau class). The Clopper–Pearson intervals do not account for the dependence between the five evaluations of one patient and are 1.3 to 1.6 times narrower than the clustered intervals in Schatzker I, II, III and VI; in types IV and V the clustered interval is the narrower of the two because the patient-level outcome is close to degenerate there, which is why the per-patient counts are tabulated. This is stated in Sections 2.5 and 4.9.

class	n_patient_evaluations_5seeds	correct	recall	recall_ci_low	recall_ci_high	precision	predicted_as_I	predicted_as_II	predicted_as_III	predicted_as_IV	predicted_as_V	predicted_as_VI
I	130	74	0.5692	0.4795	0.6557	0.4229	74	27	9	8	4	8
II	170	73	0.4294	0.3539	0.5074	0.5448	39	73	28	6	8	16
III	60	12	0.2	0.1078	0.3233	0.1846	19	23	12	3	2	1
IV	50	2	0.04	0.0049	0.1371	0.0455	20	1	6	2	10	11
V	65	7	0.1077	0.0444	0.2094	0.1795	9	3	4	14	7	28
VI	165	119	0.7212	0.6462	0.7881	0.6503	14	7	6	11	8	119

Table S6. Data integrity findings and the action taken for each (12 rows).

One row per finding. Columns give the finding, the patient it concerns where applicable, the detail, and the action taken in this study. The findings are: four conflicts between the image-level .mat label and the metadata spreadsheet; four patients whose images carry different labels because both knees were imaged; two images whose segmentation mask dimensions differ from the radiograph; one inconsistency between the data descriptor and the deposit in how coronal CT availability is documented; and one cohort age that does not reproduce from the released metadata. Every action taken is recorded so that the analysis can be reconstructed from the public release.

finding	patient_id	detail	action_taken
label conflict (.mat vs metadata spreadsheet)	230.0	image labels I vs metadata IV	image-level .mat label used; both reported
label conflict (.mat vs metadata spreadsheet)	235.0	image labels VI vs metadata I	image-level .mat label used; both reported
label conflict (.mat vs metadata spreadsheet)	238.0	image labels IV vs metadata I	image-level .mat label used; both reported
label conflict (.mat vs metadata spreadsheet)	246.0	image labels V vs metadata IV	image-level .mat label used; both reported
labels differ between images of one patient (bilateral acquisition)	92.0	labels I|NTPF	image-level labels used; NTPF images excluded from the six-class task
labels differ between images of one patient (bilateral acquisition)	112.0	labels NTPF|V	image-level labels used; NTPF images excluded from the six-class task
labels differ between images of one patient (bilateral acquisition)	133.0	labels NTPF|VI	image-level labels used; NTPF images excluded from the six-class task
labels differ between images of one patient (bilateral acquisition)	147.0	labels II|NTPF	image-level labels used; NTPF images excluded from the six-class task
segmentation mask dimensions differ from the radiograph	47.0	047_im1: image 2517x1356, mask not applicable	excluded from mask-dependent inputs (ROI, background)
segmentation mask dimensions differ from the radiograph	81.0	081_im1: image 2819x1290, mask not applicable	excluded from mask-dependent inputs (ROI, background)
coronal CT documented inconsistently between descriptor and deposit		data descriptor lists a coronal CT for every image; the deposit marks the field optional; 150/186 present	reported
reported cohort age not reproducible from the released metadata		descriptor 45.88 +/- 17.54 (n=186); recomputed 43.60 +/- 17.85; sex counts and class percentages match exactly	reported

Table S7. The non-anatomical comparator broken into feature blocks (12 rows).

One row per task and feature block. The pre-specified comparator pools twelve columns of three provenances: the clinical record (age, laterality, number of images, coronal CT availability), the acquisition (maximum height, maximum width, the larger of the two, whole-leg and landscape flags) and the annotation (mask area fraction). Only the acquisition columns describe something a network could also read off the pixels. Each block was fitted on the same partitions as the pooled model, and the columns give the block, whether it is the prespecified comparator, its balanced accuracy over five seeds, and the paired difference between the image model and that block. Two results are worth reading directly: on the binary task coronal CT availability alone, a single indicator invisible to the network, reaches 0.785 against the network's 0.833; and on the six-class task the clinical-record block alone exceeds the pooled twelve-column comparator, so the prespecified

comparator was not the strongest non-anatomical predictor available. The blocks were separated after the main results were known and are reported as a sensitivity analysis, not as a replacement for the comparator fixed in advance.

Supplied as a separate machine-readable file (Table_S7.csv, and as a sheet in Supplementary_Tables.xlsx).
